# De Novo Design of Integrin α5β1 Modulating Proteins to Enhance Biomaterial Properties

**DOI:** 10.1002/adma.202500872

**Published:** 2025-06-09

**Authors:** Xinru Wang, Jordi Guillem‐Marti, Saurav Kumar, David S. Lee, Daniel Cabrerizo‐Aguado, Rachel Werther, Kevin Alexander Estrada Alamo, Yan Ting Zhao, Adam Nguyen, Irina Kopyeva, Buwei Huang, Jing Li, Yuxin Hao, Xinting Li, Aritza Brizuela‐Velasco, Analisa Murray, Stacey Gerben, Anindya Roy, Cole A. DeForest, Timothy Springer, Hannele Ruohola‐Baker, Jonathan A. Cooper, Melody G. Campbell, Jose Maria Manero, Maria‐Pau Ginebra, David Baker

**Affiliations:** ^1^ Department of Biochemistry University of Washington Seattle WA USA; ^2^ Institute for Protein Design University of Washington Seattle WA USA; ^3^ Biomaterials, Biomechanics and Tissue Engineering Group Department of Materials Science and Engineering Universitat Politècnica de Catalunya – BarcelonaTech (UPC) Barcelona Spain; ^4^ Networking Research Centre of Bioengineering Biomaterials and Nanomedicine (CIBER‐BBN) Institute of Health Carlos III Madrid Spain; ^5^ Division of Basic Sciences Fred Hutchinson Cancer Center Seattle WA USA; ^6^ Department of Genome Sciences University of Washington Seattle WA USA; ^7^ Oral Health Sciences School of Dentistry University of Washington Seattle WA USA; ^8^ Institute for Stem Cell and Regenerative Medicine University of Washington Seattle WA USA; ^9^ Graduate Program in Biological Physics, Structure and Design University of Washington Seattle WA USA; ^10^ Department of Bioengineering University of Washington Seattle WA USA; ^11^ Program in Cellular and Molecular Medicine Boston Children's Hospital Boston MA 02115 USA; ^12^ Department of Pediatrics Harvard Medical School Boston MA 02115 USA; ^13^ Department of Biological Chemistry and Molecular Pharmacology Harvard Medical School Boston MA 02115 USA; ^14^ DENS‐ia Research Group Faculty of Health Sciences Miguel de Cervantes European University Valladolid Spain; ^15^ Department of Chemical Engineering University of Washington Seattle WA USA; ^16^ Department of Chemistry University of Washington Seattle WA USA; ^17^ Molecular Engineering & Sciences Institute University of Washington Seattle WA USA; ^18^ Institute for Bioengineering of Catalonia Barcelona Institute of Science and Technology Barcelona Spain; ^19^ Howard Hughes Medical Institute University of Washington Seattle WA USA; ^20^ Present address: LILA Biologics Seattle WA USA; ^21^ Present address: Human Biology Division Fred Hutchinson Cancer Center Seattle WA USA

**Keywords:** biomaterial, de novo protein design, hydrogel, integrin α5β1, regenerative medicine, RGD, titanium

## Abstract

Integrin α5β1 is crucial for cell attachment and migration in development and tissue regeneration, and α5β1 binding proteins can have considerable utility in regenerative medicine and next‐generation therapeutics. We use computational protein design to create de novo α5β1‐specific modulating miniprotein binders, called NeoNectins, that bind to and stabilize the open state of α5β1. When immobilized onto titanium surfaces and throughout 3D hydrogels, the NeoNectins outperform native fibronectin (FN) and RGD peptides in enhancing cell attachment and spreading, and NeoNectin‐grafted titanium implants outperformed FN‐ and RGD‐grafted implants in animal models in promoting tissue integration and bone growth. NeoNectins should be broadly applicable for tissue engineering and biomedicine.

## Graphical abstract

NeoNectins are de novo‐designed miniproteins that selectively bind and stabilize the extended open form of integrin α5β1. When grafted onto biomaterials including hydrogel and titanium, they promote cell attachment and spreading in vitro and promoting tissue integration and bone growth in animal models, demonstrating broad potential for regenerative medicine and biomaterials.

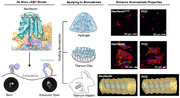

## 1 Introduction

**Figure 1:**
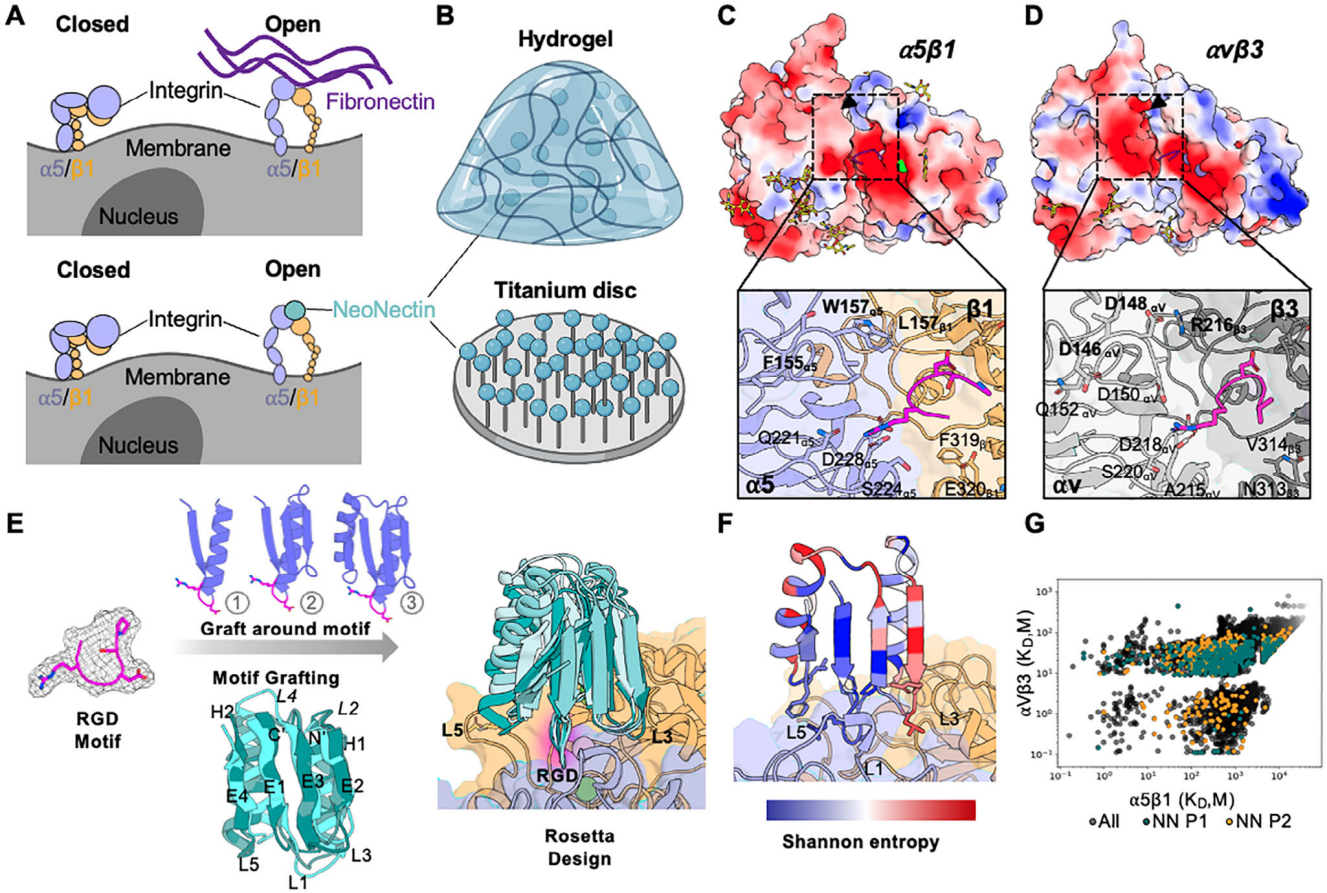
α5β1 binder design strategy. A) Schematic representation of the structure of the inactive, apo‐integrin α5β1, Fibronectin‐bound, active integrin α5β1(top panel), and NeoNectin‐bound (blue circle) integrin α5β1 (bottom panel). Integrin α5 subunit is shown as slate blue and β1 subunit is shown as orange. B) Schematic of designed NeoNectin in biomaterial applications. C,D) Specificity design challenge highlighted by the similar electrostatic potential of integrins α5β1, αvβ3 (structures are from complexes with their cognate ligand peptides; PDB:4WK2 and 1L5G, respectively). Main differences are highlighted with arrows. Glycan molecules are shown as yellow sticks. Zoomed‐in views of the RGD binding interfaces of α5β1 and αvβ3 are shown below. E) Design strategy for α5β1 specific NeoNectin. F) Computational model of a designed α5β1 binder colored by site saturation mutagenesis results. The NeoNectin parent design 1 was colored by positional Shannon entropy, using a gradient from blue to red, with blue indicating positions of low entropy (conserved) and red those of high entropy (not conserved). G) Site Saturation Mutagenesis analysis of NeoNectin parent designs was sorted by FACS in the presence of α5β1 at different concentrations and the affinity of each variant was calculated. The affinity of each variant of NN parent design 1 (NN P1) and parent design 2 (NN P2) were highlighted as green and orange circles, respectively. The upper left corner are variants specific to α5β1.

Integrin α5β1 is one of the principal receptors for fibronectin (FN), a major component of extracellular matrix (ECM) component that is extensively expressed in various cells and tissues. The interactions between α5β1 and FN are vital for cell attachment and migration, making them integral to various stages of development and tissue regeneration, notably in wound healing, bone regeneration, and stem cell therapy.^[^ ^1^, ^2^, ^3^, ^4^ ^]^ However, the clinical use of full‐length FN or its main interacting RGD (Arg‐Gly‐Asp) motif on biomaterials for regenerative purposes has been challenging. Full‐length FN, typically derived from human plasma, poses challenges for large‐scale production and is vulnerable to protease cleavage.^[^ ^5^, ^6^ ^]^ Conversely, while the RGD peptide can be easily manufactured and is widely used in biomaterial coatings, it does not elicit the desired cellular responses and does not consistently enhance bone formation in vivo,^[^ ^7^, ^8^, ^9^ ^]^ perhaps because of the low affinity of RGD peptides to α5β1 compared to FN^[^ ^10^ ^]^ or because of the broad reactivity with the eight RGD/binding integrins.^[^ ^11^, ^12^ ^]^ The α5β1, α8β1, αvβ1, αvβ3, αvβ5, αvβ6, αvβ8, and αIIbβ3^[^ ^13^ ^]^ integrins all have the conserved RGD binding pocket with nearby glycan molecules making the design of integrin specific peptides challenging^[^ ^11^, ^12^, ^14^ ^]^ (**Figure** **1**B–F). Integrin α5β1 undergoes a large conformational change from the inactive closed state to the active open state when bound to FN.^[^ ^15^, ^16^ ^]^

We reasoned that de novo protein design could enable the creation of small, stable, and easy to manufacture α5β1 binders that specifically activate α5β1 on biomaterials (Figure 1A,B). We set out to design α5β1 protein binders that can bind the interface between α5 and β1 subunits, including the RGD‐binding pocket and adjacent regions, to stabilize the active conformation of α5β1. Due to their in silico origin and outstanding capacity to enhance cell adhesion, we call them NeoNectins (NN).

## 2 Results

### 2.1 Computational Design

Our goal was to design proteins to bind the groove formed between the α5 and β1 integrin subunits, specifically stabilizing the active extended open (EO) conformation of α5β1 (Figure 1A). While the RGD binding site is highly conserved between RGD binding integrins, a nearby unique hydrophobic pocket formed by Trp157_α5_, Phe155_α5_, and L157_β1_ offers opportunities for targeting α5β1 specifically (Figure 1C). In contrast, the corresponding residues of the close structural homolog, αvβ3 are hydrophilic (S146_αv_, D148_αv,_ and R216_β3,_ Figure 1D). Ferredoxin scaffolds, with their two helices and four beta strands, offer high stability and are ideal for loop presentation (Figure 1E). We explored both the stepwise building up of ferredoxin^[^ ^17^ ^]^ starting from the RGD loop of FN extracted from an RGD/α5β1 complex structure (PDB:4WK2) or grafting the RGD loop onto computationally pre‐built ferredoxin scaffold libraries^[^ ^18^ ^]^ (Figure 1E). In both cases, Rosetta flexible backbone protein design^[^ ^18^, ^19^ ^]^ was then used to optimize the structure and the sequence of the design for shape complementarity and extensive interactions with α5β1. Designs were ranked based on Rosetta binding energy (ddG), solvent‐accessible surface area, molecular contact surface, and a deep learning‐based monomer folding metric.^[^ ^20^ ^]^ A total of 7820 designs from the first approach and 12674 from the second approach were selected for experimental characterization.

### 2.2 Experimental Characterization

**Figure 2:**
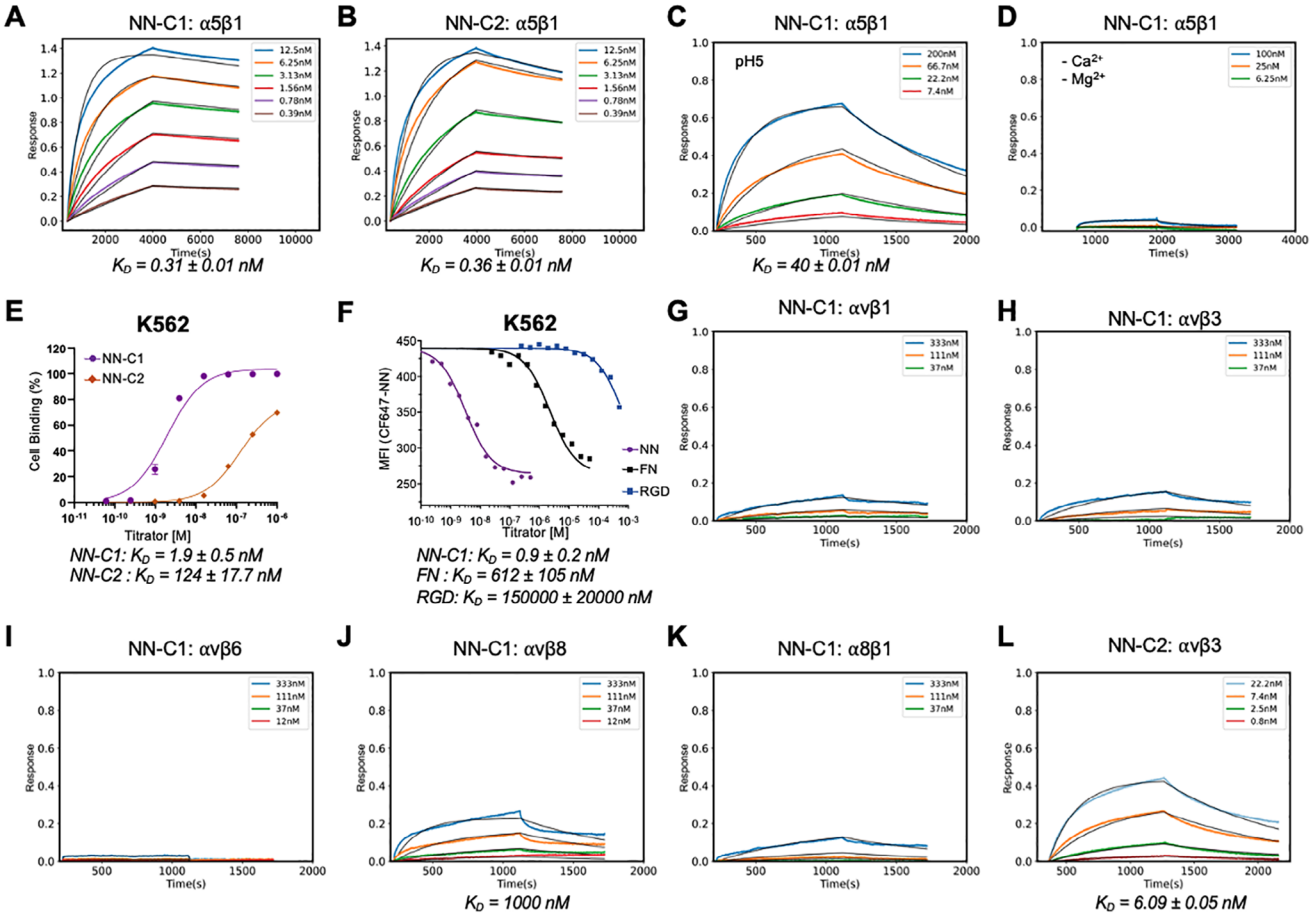
NeoNectin binds α5β1 with high affinity and specificity. A–D) BLI binding affinity traces for NeoNectin candidates 1 (NN‐C1) or 2 (NN‐C2) against the α5β1 ectodomain in integrin resting buffer (20 mm Tris, pH 7.4, 1 mm Ca^2+^, 1 mm Mg^2+^) or otherwise noted. Global kinetic fits, assuming a 1:1 binding model, are shown as black lines. E) Flow cytometry measurements of biotinylated NeoNectin candidates on K562 cells gave K_D_​ values of 1.9 nm for NN‐C1 and 124 nm for NN‐C2. F) Competing the binding of CF647 labeled NN‐C1 by NN‐C1, FN, and RGD peptide on K562 cells gave K_D_ values of 0.9 ± 0.2, 612 ± 105, and 150 000 ± 20,000 nm respectively (see Methods for calculating K_D_ values). G–K) BLI binding affinity traces for NN‐C1 against αvβ1, αvβ3, αvβ6, αvβ8, and α8β1 ectodomain in integrin resting buffer. L) BLI binding affinity traces for NN‐C2 against integrin αvβ3 in the resting buffer.

Synthetic oligonucleotides encoding the designs were cloned into a yeast surface‐expression vector. Yeast cells displaying the designed proteins were incubated with biotinylated α5β1 ectodomain and several rounds of fluorescence‐activated cell sorting (FACS) were used to enrich those that bound α5β1. The starting and enriched populations at each round were deep sequenced, and the frequency of each design in the starting population and after each sort was determined; this was used to estimate binding dissociation constants (K_D_) for each design.^[^ ^18^ ^]^ For the 16 most enriched designs, we generated site saturation mutagenesis libraries (SSMs), in which every residue was substituted with each of the 20 amino acids, and sorted the SSMs in the presence of α5β1 or αvβ3, an α5β1 homolog, at different concentrations and the affinity of each variant was calculated (Figure 1F,G; Figure S1A–F, Supporting Information). Substitutions at the RGD site in the SSMs were not tolerated for 8 out of 16 designs, as expected given the central role this motif plays in mediating integrin α5β1 and design interactions (Figure S1E,F, Supporting Information). Between 5–8 substitutions at designed interacting loop 3 and loop 5 that increased the apparent binding affinity were combined in small libraries which were sorted under stringent conditions (incubation with 0.2 nm biotinylated α5β1 followed by washing and overnight dissociation), yielding 20 optimized designs which were expressed in *E. coli* and purified (Figure S1G,H, Supporting Information). Biolayer interferometry (BLI) showed that 5 designs bind to α5β1 with affinities ranging from subnanomolar to nanomolar K_D_ (**Figure** **2**A,B; S1I–K, Supporting Information).

### 2.3 NeoNectin Binds to Integrin α5β1 Tightly and Specifically

NeoNectin candidates 1 (NN‐C1) and 2 (NN‐C2) bind most tightly to integrin α5β1 among the tested designs, with similar K_D_ of 0.31 and 0.36 nm in integrin resting buffer (pH 7.4, 1 mm Ca^2+^/Mg^2+^), measured by BLI (Figure 2A,B). Both candidates dissociate slowly, with minimal dissociation observed within an hour. This binding is dependent on the RGD binding to the Metal Ion‐Dependent Adhesion Site (MIDAS) that is sensitive to pH, metal content, and RGD residues (Figure 2C,D; Figure S1L, Supporting Information). Interestingly, NN‐C1 exhibits 65‐fold stronger affinity than NN‐C2 in binding to wild‐type K562 lymphoblast cells, in which α5β1 is the only expressing RGD‐interacting integrin (Figure 2E). The difference between the K_D_ toward integrin α5β1 on cells could be potentially explained in their capacity in stabilizing the extended open (EO) conformation of integrin (described below). We then compared the relative affinity of FN and RGD peptide to NN‐C1 by competing unlabeled NN‐C1, FN, or RGD peptide with CF647‐ labeled NN‐C1 on K562 cells in L15 medium with 1% BSA. FN or RGD peptide showed much lower binding affinities than NN‐C1, with IC50 values of 612 and 150 000 nm, respectively (Figure 2F). Thus, NN‐C1 binds to α5β1 680 times more tightly than FN and 167 000 times more tightly than RGD peptide on cells. A cyclic peptide ACRGDGWCG (cyclization through underlined cysteines), previously developed using phage display libraries,^[^ ^21^, ^22^ ^]^ was found to exhibit a K_D_ of 3700 ± 500 nm to intact cells in L15 medium and 200 ± 16 nm with 1 mm Mn^2^⁺ present.^[^ ^23^ ^]^

We then tested if the NeoNectin candidates bind to integrin α5β1 specifically. There was little binding toward other RGD binding integrins for NN‐C1, measured by BLI and fluorescence polarization assays (Figure 2G–K; Figure S2B–I, Supporting Information). However, NN‐C2 also binds to αvβ3 with a K_D_ of 6.1 nm, indicating less specificity (Figure 2L). We then focused on if the NN‐C1 binds MCF10A Cas^mSc^ cells^[^ ^24^ ^]^ in an α5β1‐dependent manner. MCF10A Cas^mSc^ cells is a human mammary epithelial cell line expressing endogenously tagged Cas with mScarlet to mark the integrin positive adhesion.^[^ ^25^, ^26^ ^]^ Besides α5β1, MCF10A cell line also expresses other RGD binding integrins including αvβ1, αvβ3, αvβ5, and αvβ6.^[^ ^27^, ^28^ ^]^ We first incubated MCF10A Cas^mSc^ cells^[^ ^24^ ^]^ in presence or absence of 200 nm α5β1‐specific antibody mAb16 followed by incubation of 10 nm NN‐C1. The cells were then imaged by Total Internal Reflection Fluorescence Microscopy. NeoNectin was only bound to MCF10A when cells were not pretreated with mAb16, suggesting it is specific for cellular α5β1 (Figure S1N, Supporting Information).

### 2.4 NeoNectin‐Bound Integrin α5β1 Favors Extended Conformations

**Figure 3:**
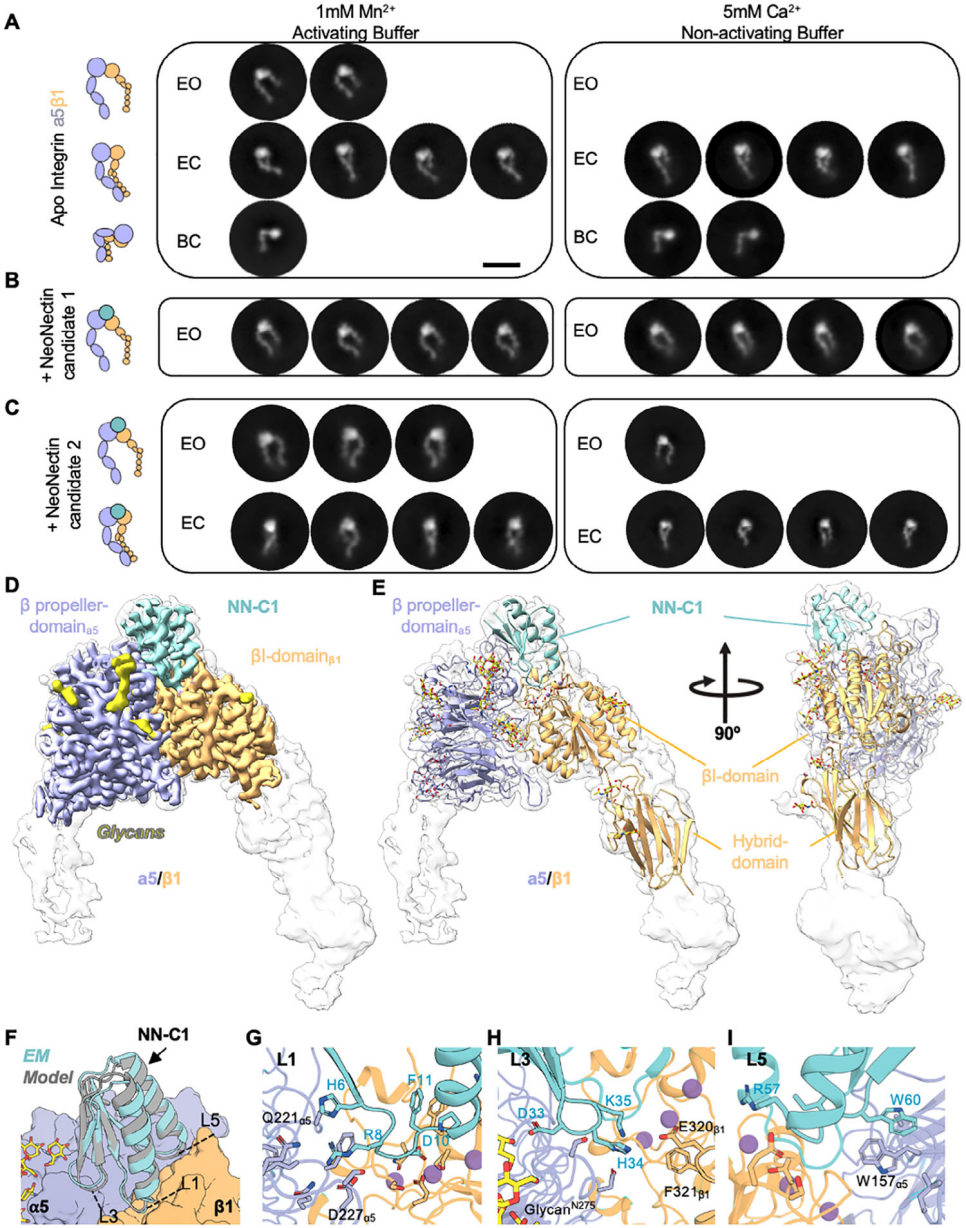
Structural characterization of integrin α5β1:NeoNectin complexes. A–C) Representative 2D negative stain class averages of α5β1 alone, in presence of NN‐C1 or NN‐C2 in activating (1 mm Mn^2+^) and non‐activating (5 mm Ca^2+^) buffer conditions. Integrins are categorized into three canonical conformations: extended open (EO), extended closed (EC), and bent closed (BC). The number of classes shown is representative of the number of total particles in that conformation. D) Cryo‐EM map of α5β1 bound to NeoNectin. The sharpened, locally refined map is shown in color, superimposed with the unsharpened map in semi‐transparent white. The color code is as follows: α5 (lavender), β1 (light orange), Neonectin (turquoise), coordinated cations (plum), and glycans (yellow). E) Two views of the ribbon model of α5β1 bound to NeoNectin displayed within the unsharpened density shown in A). F) An overlay of the NN‐C1 designed model (gray) and the experimentally determined model (turquoise). G) Close‐up of NN‐C1 Loop1 (L1, ^6^HRGDFP)^[^ ^12^ ^]^ and α5β1. R8 and D10 directly interact with α5β1 and other residues contribute to stabilizing the loop. H) Close‐up of NN‐C1 Loop3 (L3, ^33^DHK)^[^ ^35^ ^]^ and α5β1 interface. I) Close‐up of NN‐C1 Loop5 (L5, ^57^RGLW)^[^ ^60^ ^]^ and α5β1 interface.

To investigate the effects of NeoNectin candidates on α5β1 conformation and the molecular basis of the interactions, we used negative stain Electron Microscopy (nsEM) and cryogenic Electron Microscopy (cryo‐EM). Many integrins, including α5β1 are known to undergo drastic conformational changes that are linked to activation state. In vitro, it has been established that high Ca^2+^ (5 mm) stabilizes the low‐affinity, closed headpiece conformation, while 1 mm Mn^2+^ stabilizes the high‐affinity conformation with an open headpiece and extended legs^[^ ^29^ ^]^ (**Figure** **3**A). Using nsEM we found that in both cation conditions, NN‐C1 binds and favors the EO conformation of integrin α5β1 (Figure 3B) while NN‐C2 bound α5β1 showed a mixture of EO and EC conformation (Figure 3C). This suggests that the observed differences in cell binding affinity between NN‐C1 and NN‐C2 may result from the various ability to stabilize EO conformation (Figure 2F).

Next, we determined the structure of α5β1 in complex with NN‐C1to a local resolution of 3.2 Å and an overall resolution of 3.3 Å by cryo‐EM (Figure 3D,E; Figure S4A and Table S1, Supporting Information). As expected, the dominant class (≈88% of integrin particles) shows the α5β1 headpiece in an open conformation (Figure 3B,D; Figure S5A and Table S1, Supporting Information) with NeoNectin making interactions and inducing downstream large‐scale structural rearrangements of α5β1 similar to the native fibronectin ligand.^[^ ^16^ ^]^ The interaction between NeoNectin and α5β1 in the experimentally determined cryo‐EM model is consistent with the designed model in that it centers around three short loops: L1 (Gly7, Arg8, Gly9, Asp10, Phe11, Pro12), L3 (Asp33, His34, Lys35), and L5 (Gly58, Ile59, Trp60) (Figure 3F–I; Figure S6A, Supporting Information). The RGD motif encoded by L1 binds α5β1 via the same entities as the RGD motif in FN or RGD peptide.^[^ ^16^, ^30^ ^]^ Specifically, Gln221_α5_ and Asp227_α5_ bind Arg and β1‐coordinated MIDAS cation coordinates Asp (Figure 3G). L3 further stabilizes the interaction through interactions with both α5 and β1 subunits: His34_NN‐C1_ and Lys35_NN‐C1_ form a salt bridge triad with Glu320_β1_ and His34_NN‐C1_ forms an additional interaction with Leu225_α5_ backbone (Figure 3G). While FN also forms a salt bridge with Glu320_β1_ that was suggested to be important for FN‐induced headpiece opening through Arg1445_FN_ and Tyr1446_FN_^[^ ^16^ ^]^ (Figure S6J, Supporting Information), mutating His34_NN‐C1_ and Lys35_NN‐C1_ to Gly did not prevent headpiece opening (Figure S6B, Supporting Information). NeoNectin L5 Trp60 interacts via shape complementarity with residues in both integrin subunits, including a pi‐pi stacking interaction with Trp157_α5_. It has been shown previously that Trp157_α5_ introduces α5β1 specificity into RGD cyclic peptides (Figure S6I, Supporting Information); however, Trp157_α5_ is not required for binding to FN.^[^ ^12^, ^16^, ^31^ ^]^ When mutated to Ala, the NeoNectin W60A variant still favors the open conformation of α5β1, thus suggesting it does not influence headpiece opening directly (Figure S6B, Supporting Information), but instead contributes to binding affinity and specificity.

Next, we aimed to characterize the interactions of α5β1 with NN‐C2, which shares 50% sequence identity with NN‐C1 (Figure S1M, Supporting Information). As expected from the nsEM, in cryo‐EM, we observed both the open (67%) and closed headpiece (33%) conformations (Figure S5A, Supporting Information). We resolved the open headpiece complex to a resolution of 4.0 Å overall and 3.6 Å locally and the closed headpiece complex to a resolution of 3.0 Å overall and 3.0 Å locally (Figures S5B,C, S6C–E, and Table S1, Supporting Information). The contacts made between NN‐C2 and α5β1 were strikingly similar to the contacts made by NN‐C1. However, while Mn^2^⁺ is observed at the ADMIDAS site in the open α5β1:NN‐C2 complex (Figure S6F, Supporting Information), no ADMIDAS ion is observed in the closed α5β1:NN‐C2 complex (Figure S6G, Supporting Information). In the closed α5β1:NN‐C2 complex, the additional salt bridges are made by Arg13_NN‐C2_ and Asp259_β1_ and Asp138_β1_. In contrast, the side chain of Arg13_NN‐C2_ is not resolved in the EO complex, suggesting conformational flexibility. This is consistent with the importance of ADMIDAS engagement in stabilizing the EO conformation.^[^ ^16^ ^]^

### 2.5 Soluble NeoNectin Inhibits α5β1‐Mediated Cellular Behaviors

**Figure 4:**
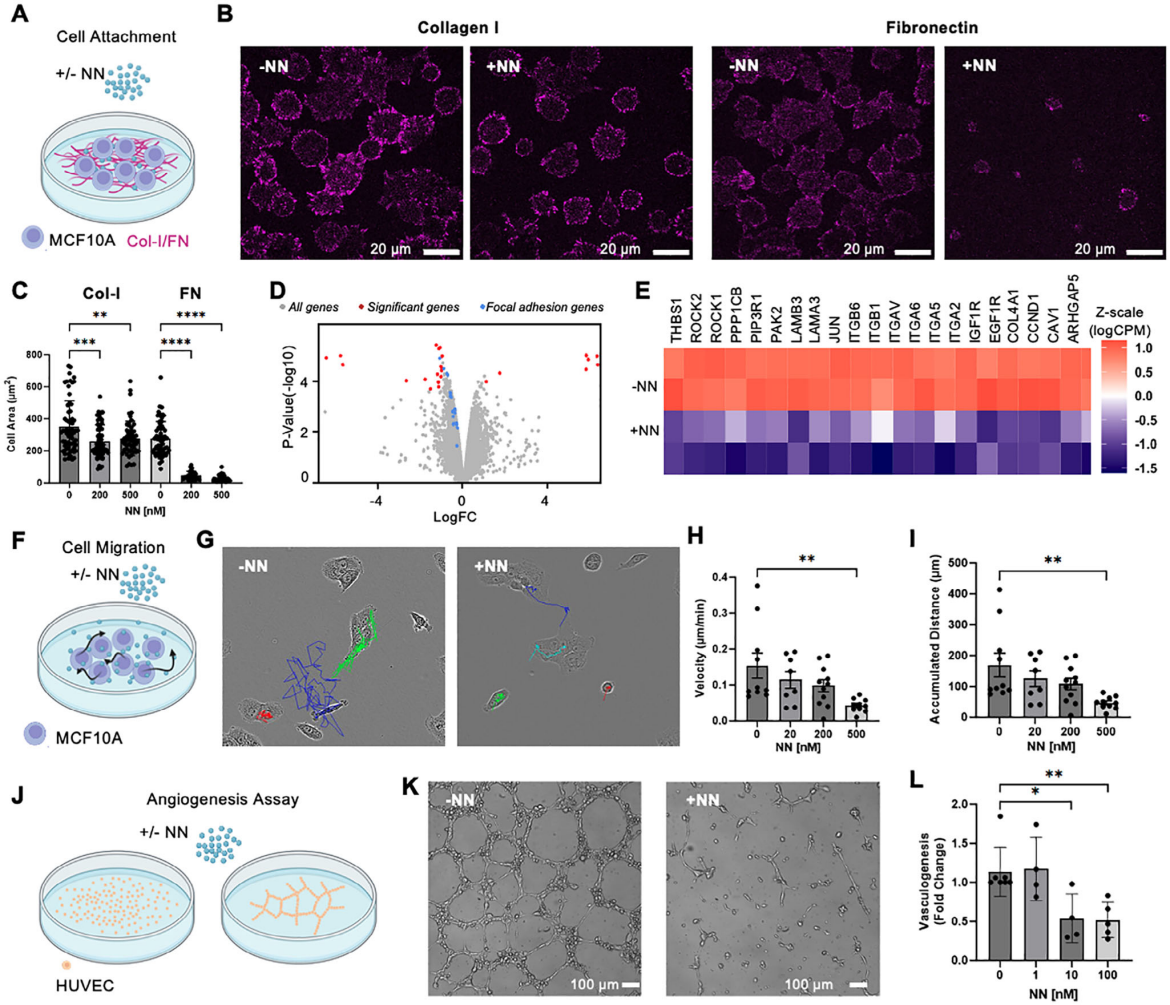
Soluble NeoNectin inhibits α5β1‐mediated cell spreading and migration. A) Schematic of the experimental design monitoring MCF10A cell attachment in presence of soluble NeoNectin on collagen I or FN‐coated surface. B) Confocal imaging of MCF10A cells plated on collagen I or FN coated surface in presence of soluble NeoNectin for 30 min. The scale bar is 20 µm. C) Quantification of cell area in B). D) RNA‐seq results from MCF10A cells plated on fibronectin surface in presence or absence of 500 nm NeoNectin for four h, with genes related to the focal adhesion pathway highlighted in blue, genes significantly affected highlighted in red. (E) Heat map representation of genes (as Z‐score of logCPM) involved in focal adhesion pathway. ‐NN: Cells spread on FN‐coated surface. +NN: Cells spread on FN‐coated surface in the presence of 500 nm NeoNectin. F) Schematic of the experimental design monitoring MCF10A cell migration with/without soluble NeoNectin. G) Trajectories of individual cells tracked over an 18‐hour imaging period in presence of 0 or 500 nm NeoNectin. H) Quantification of cell velocity in µm/min of individual cells from G) and Figure S6C (Supporting Information). I) Quantification of accumulated traveled distance of individual cells from G) and Figure S6C (Supporting Information). J) Schematic of the tube formation assay. K) Representative decrease in vascular stability by 10 nm soluble NeoNectin. Soluble NeoNectin was added to HUVEC cells at 0, 1, 10, and 100 nm. Vascular stability was analyzed after 12 h. L) The number of nodes, meshes, and tubes was quantified using an angiogenesis analyzer plug‐in in ImageJ. The scale bar is 100 µm. Statistical significance was analyzed using One‐way Anova Bonferroni's multiple comparison test. All experiments have at least three biological replicates.

Due to the exceptional behavior of NN‐C1, we will refer to it as “NeoNectin” throughout the remainder of the paper. Integrin α5β1 regulates cellular events such as cell attachment, cell migration, and tubulogenesis through interaction with FN found within the ECM. Both NeoNectin and FN bind to the RGD binding site of α5β1. Because of the high affinity and specificity of NeoNectin toward α5β1, we hypothesized that NeoNectin in solution can inhibit α5β1‐FN interaction dependent cellular responses as mentioned above. We first tested the effect of soluble NeoNectin on inhibiting spreading of MCF10A epithelial cells, which can migrate on ECM‐containing FN and/or collagen.^[^ ^24^ ^]^ We first plated MCF10A cells on either collagen‐ or FN‐precoated glass surfaces and then treated the cells with NeoNectin at different concentrations for 30 min prior to washes. The remaining cells were imaged using fluorescent microscopy (**Figure** **4**A). Soluble NeoNectin dramatically reduced cell attachment to FN‐coated surfaces but not to collagen‐coated surfaces, suggesting that NeoNectin does not interact with integrins specific for collagen‐binding (Figure 4B,C). Similar effects were also observed in A549 adenocarcinoma human alveolar basal epithelial cells cultured on FN‐grafted titanium discs or discs grafted with the cell attachment site (CAS) fragment from FN, but not laminin‐ (LAM, responsible for binding to integrins α1β1, α2β1, α3β1, α6β1, α7β1, and α6β4 without involving RGD) and vitronectin‐grafted (VTN; responsible for αvβ3 integrin binding) titanium discs (Figure S7A,B, Supporting Information).

To investigate the effects of soluble NeoNectin on gene expression, we plated MCF10A cells on FN‐coated plates and treated them with/without NeoNectin. After 4 h, we harvested the MCF10A cells and analyzed bulk transcript levels using RNA sequencing. Pathway enrichment analysis determined that NeoNectin treatment significantly down‐regulated focal adhesion‐related genes (THBS1, ROCK2, ROCK1, PPP1CB, PIP3R1, PAK2, LAMB3, LAMA3, JUN, COL4A1, CCND1, CAV1, and ARHGAP5), integrin subunits (αv, α2, α5, α6, β1, and β6), and growth factor receptor tyrosine kinases (IGF1R and EGF1R) expression (*p*‐value = 3.5 × 10^−7^) (Figure 4D,E).

Next, we evaluated the effects of NeoNectin on inhibiting epithelial cell migration by culturing the cells on FN‐coated surfaces and monitoring their positions every 10 min for 18 h after treatment with NeoNectin at different concentrations (Figure 4F). Cell migration is mediated by FN binding and recycling of integrins,^[^ ^32^, ^33^ ^]^ and hence, we expect the soluble NeoNectin to inhibit this process. Indeed, we observed a decrease in cell velocity and distance covered at all concentrations tested (Figure 4H,I; Figure S7C, Supporting Information).

We evaluated the capacity of NeoNectin to inhibit angiogenesis using human umbilical vein endothelial cells (HUVECs) in a tube formation assay (Figure 4J). Soluble NeoNectin was seeded with HUVECs at 0.1 to 100 nm or with PBS as control for 12 h before images were taken. Tube formation was significantly attenuated at 10 nm of NeoNectin (Figure 4K,L).

### 2.6 Immobilized NeoNectin in Hydrogel Promotes Cell Attachment and Spreading

**Figure 5:**
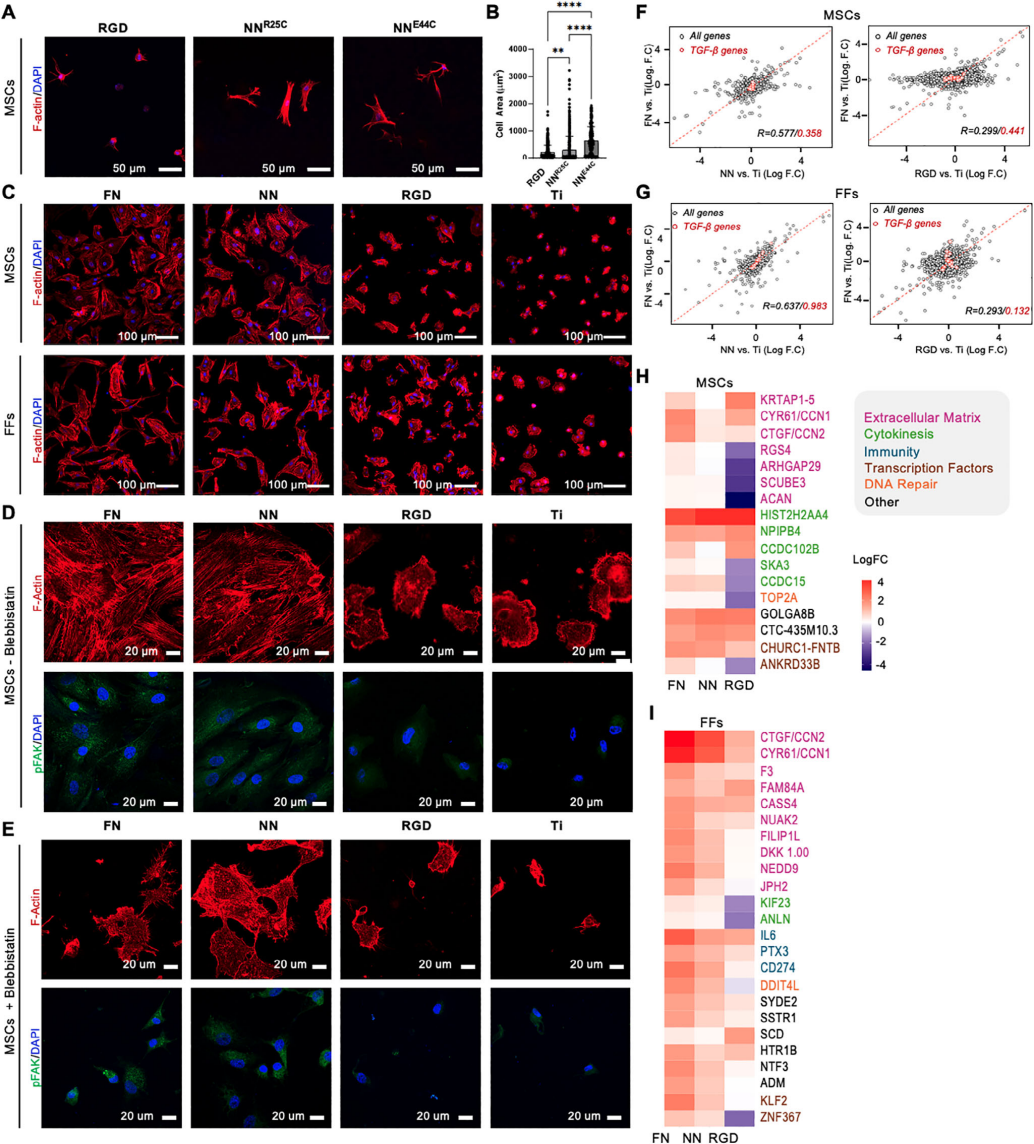
Immobilized NeoNectin stimulates FN‐like cells spreading in 3D and 2D cultures. A) Representative immunofluorescence images of MSCs after 5 days of 3D culture within the different functionalized hydrogels. Adhesion modifications were included at 1 mm concentration. The scale bar denotes 50 µm. RGD: CRGDS. Nuclei are stained in blue, and Actin cytoskeleton in red. B) Quantification of cell spread area in A). One Way ANOVA, Tukey's Post‐hoc Test. **p < 0.01, ***p < 0.001, ****p < 0.0001. C) Representative immunofluorescence images of MSCs (top) and FFs (bottom) after 4 h of adhesion on the different functionalized surfaces. FN and NeoNectin were covalently linked through free amines. The RGD peptide (Cys‐(Ahx)3‐GRGDS) was covalently attached through the Cys. The scale bar denotes 100 µm. Nuclei are stained in blue, and Actin cytoskeleton in red. D) Representative immunofluorescence images of MSCs to detect phosphorylation of FAK. Nuclei are stained in blue, Actin cytoskeleton is stained in red, and pFAK (Tyr397) is in green. The scale bar denotes 20 µm. E) Representative immunofluorescence images of MSCs to detect phosphorylation of FAK after treatment with blebbistatin. Nuclei are stained in blue, Actin cytoskeleton is stained in red, and pFAK (Tyr397) in green. The scale bar denotes 20 µm. F,G) Scatterplots comparing relative gene expression (fold change of FN‐, NN‐, or RGD‐grafted Ti over bare Ti) for MSCs F) and FFs G). FN‐grafted samples are on the y‐axis; NN‐ (left) or RGD‐treated (right) samples on the *x*‐axis. Similarities of whole transcriptome (black) and TGF‐β pathway genes (red) with FN‐grafted samples were assessed by Pearson correlation. H,I) Heat map representation of top differentially expressed genes compared to cells spread on bare titanium surface (Log of Fold Change (LogFC) > 1.5 or < −1.5 in MSCs). FN: Cells spread on FN‐grafted titanium surface. NN: Cells spread on NeoNectin‐grafted titanium surface. RGD: Cells spread on RGD peptide‐grafted titanium surface. ECM: Extracellular Matrix. TF: Transcription Factors.

Because NeoNectin binds integrin α5β1 more tightly than RGD and favors the active EO conformation (Figure 3B), we hypothesized that NeoNectin could enhance the properties of biomaterials for stem cell encapsulation. We first investigated whether NeoNectin could enhance human mesenchymal stem cells (MSCs) adhesion and spreading in a 3D hydrogel (Figure S8A, Supporting Information). Two NeoNectin variants with cysteines at solvent‐exposed locations (Figure S8B, Supporting Information; NeoNectin^R25C^, and NeoNectin^E44C^) were covalently tethered within poly(ethylene glycol) (PEG)‐based material via the radical thiol‐ene photopolymerization reaction.^[^ ^34^, ^35^, ^36^ ^]^ MSCs encapsulated for 5 days with the NeoNectin^R25C^ or NeoNectin^E44C^ variants at a 0.5 or 1 mm concentration displayed significantly higher spread areas with robust stress fiber formation compared to the hydrogels grafted with equimolar concentrations of RGD. The RGD condition displayed advanced protrusions, but very few stress fibers (**Figure** **5**A,B); the cellular eccentricity was not statistically significant among the conditions (Figure S8E, Supporting Information). At a concentration of 0.5 mm, MSCs encapsulated in the RGD condition remained rounded with very few protrusions, but those in the NeoNectin conditions were able to spread, resulting in significantly higher cell area and eccentricity, despite the lower availability of the adhesive moieties (Figure S8C–G, Supporting Information). Thus, NeoNectin‐modified PEG hydrogels effectively support the growth and function of encapsulated human cells.

### 2.7 Immobilized NeoNectin on Titanium Discs Promotes Cell Attachment and Spreading

We then evaluated the behavior of bone tissue cells on NeoNectin‐grafted titanium surfaces, one of the most commonly used materials in implantology. First, the immobilization of NeoNectin as a monolayer was confirmed by x‐ray photoelectron spectroscopy (Table S2, Supporting Information). The low variation observed across measurements further suggests nearly complete and consistent surface coverage, comparable to FN and RGD coatings in previous studies.^[^ ^37^, ^38^ ^]^ qPCR indicated high expression of integrins α5 and β1 subunits in MSCs and human foreskin fibroblasts (FFs), intermediate expression in human osteoblast (OBs) cells, and the lowest α5 and β1 expression in SaOS‐2 and MG‐63 osteosarcoma cell lines (Figure S9A, Supporting Information).

We hence cultured MSCs on NeoNectin‐grafted titanium discs and showed a completely spread morphology, similar to what we observed for MSCs cultured on FN‐grafted surfaces (Figures 5C (top); Figure S9B, Supporting Information). Actin cytoskeletal filaments were well‐developed and organized in both conditions (Figure S9C, Supporting Information). Focal adhesions were also present, as indicated by vinculin (Figure S9C, Supporting Information) and pFAK staining (Figure 5D), although to a lesser degree in NeoNectin‐grafted surfaces compared to FN‐grafted discs. Treatment with blebbistatin — a myosin II inhibitor — led to the loss of stress fibers and a marked reduction or disappearance of focal adhesions (Figure 5E). These results collectively indicate that NeoNectin engages integrin‐mediated mechanotransduction systems and activates force‐dependent signaling pathways in adhered cells. The calculated area (size of each cell), the cellular circularity^[^ ^39^ ^]^ (the ratio of area to perimeter), and the number of cells (Figure S9D, Supporting Information) attached to both surfaces presented no statistically significant differences. These data indicate that NeoNectin stimulates MSC attachment similarly to full‐length FN, consistent with the expression of α5, αv, and β1 subunits (Figure S9A, Supporting Information). In contrast, MSCs cultured on RGD‐grafted titanium discs presented a rounded shape similar to bare titanium discs (Figure 5C (top); Figure S9B, Supporting Information), although with some protrusions and a diffuse actin cytoskeleton (Figure S9C, Supporting Information). The area, the circularity, and the number of cells in these two conditions were significantly lower than on NeoNectin‐ and FN‐grafted surfaces (Figure S9D, Supporting Information). Although MSCs express similar amounts of αv and α5 subunit, the high specificity of NeoNectin toward α5β1 suggests that integrin α5β1 is primarily responsible for MSC spreading.

FFs grown on FN‐ and NeoNectin‐grafted titanium discs also showed similar morphology (Figure 5C (bottom); Figure S9B, Supporting Information), although fewer signs of focal adhesion were observed in the latter (Figure S9C, Supporting Information). Similar to what we observed for MSCs, FFs grown on RGD‐grafted and bare titanium discs presented a round morphology (Figure 5C; Figure S9B–D, Supporting Information). In contrast, other bone cells including OBs, SaOS‐2, and MG‐63 cell lines grown on NeoNectin‐grafted titanium discs presented a less spread morphology compared to FN‐grafted surfaces (Figure S9B,C, Supporting Information), in accordance with their lower levels of α5 and β1 integrins expression (Figure S9A, Supporting Information).

### 2.8 Cells Grown on NeoNectin‐ and FN‐Grafted Titanium Discs Produce Similar Gene Expression Patterns

To test whether NeoNectin affects differential gene expression similarly to FN, we harvested MSCs, FFs, and OBs grown on FN‐, NeoNectin‐, RGD‐grafted, or bare titanium discs and performed bulk RNA‐Sequencing. For both MSCs or FFs, we observed a strong correlation of gene expression when compared against bare titanium between FN and NeoNectin (Pearson R = 0.577, R = 0.637 respectively, Figure 5F,G (left)) but not between FN and RGD (Pearson R = 0.299, R = 0.293 respectively, Figure 5F,G (right)), suggesting that NeoNectin drives a transcriptional program more similar to FN than RGD. To determine if signaling was affected in a cell‐type dependent manner, we further focused on genes involved in the TGF‐β signaling pathway, an essential pathway involved in the activation of fibroblasts and the differentiation of MSCs/OBs.^[^ ^40^, ^41^ ^]^ Utilizing pathway enrichment analysis with Enrichr, we observed that FFs have upregulated TGF‐β signaling in FN and NeoNectin conditions when compared to bare titanium (p‐values: 5.7 × 10^−6^, 4.9 × 10^−4^ respectively), suggesting that both conditions have similar capabilities in signaling downstream in the TGF‐β pathway as demonstrated by a strong correlation of magnitude of gene expression (Pearson R = 0.984, Figure 5G). This result suggests that FN and NeoNectin activate FFs in a similar manner. In the case of MSCs and OBs, we found that TGF‐β signaling was not significantly affected in all conditions (Figure 5F; Figure S10A,B, Supporting Information), suggesting that they maintain their differentiation potential.

To understand cell behavior differences, we compared the top differentially expressed genes relative to bare titanium. Genes with a log2 fold change greater or less than 1.5 were analyzed. Both MSCs and FFs grown on FN‐ and NeoNectin‐grafted titanium discs showed increased expression of genes involved in ECM, cell attachment, proliferation, and survival (Figure 5H,I). Conversely, cells grown on RGD‐grafted discs showed down‐regulation of some of these genes. This is consistent with the less spread morphology of cells grown on RGD‐grafted Ti discs or hydrogel (Figure 5A,C; Figure S9B,C, Supporting Information).

### 2.9 NeoNectin‐Grafted Implants Enhance Osseointegration

**Figure 6:**
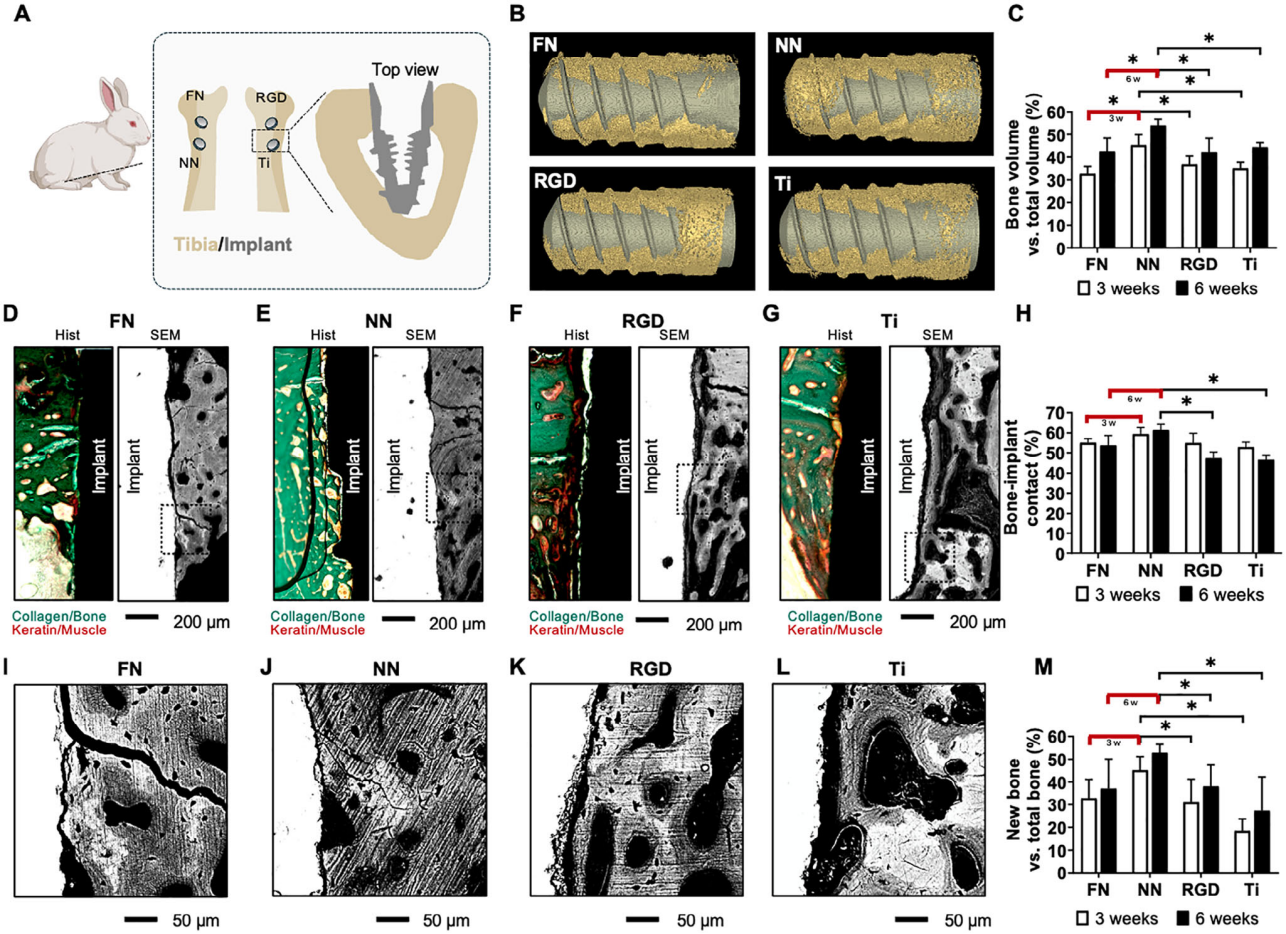
NeoNectin‐grafted titanium implants outperform FN‐grafted, RGD‐grafted, and bare titanium (Ti) implants in stimulating implant integration and bone growth. A) Schematic of the in vivo experimental procedure with rabbits. Implants were randomly inserted into the tibia of rabbits, and samples were collected for histomorphometric analyses 3 and 6 weeks after the surgical intervention. N = 7 for the 3‐week and 6‐week groups. B) Representative micro‐CT 3D reconstruction images showing bone (yellow) around the grafted or bare titanium implants (gray) 3 weeks post‐surgery. C) Calculated percentage of bone volume versus total volume (BV/TV) from micro‐CT images collected from animals at 3 weeks B) and 6 weeks groups (Figure S11A, Supporting Information) post‐surgery. Non‐parametric Mann Whitney's test (*p < 0.05). Data are presented as mean ± standard error of the mean. Direct comparisons between FN‐ and NeoNectin‐grafted titanium implants were highlighted in red for both the post‐3‐week B) and 6‐week groups (Figure S11A, Supporting Information). D–G) Representative histological staining (left) and SEM (right) images of longitudinal sections 3 weeks post‐implantation showing the implants conjugated with indicated molecules inserted into the tibia of rabbits. Bones are stained in green, and muscle in red. The scale bar denotes 200 µm. H) Calculated bone‐implant contact (BIC) percentage from SEM images D–G), and Figures S11C–F (Supporting Information). Non‐parametric Mann Whitney's test (*p < 0.05). Data are presented as mean ± standard error of the mean. Direct comparisons between FN‐ and NeoNectin‐grafted titanium implants were highlighted in red for both the post‐3‐week D–G) and 6‐week groups (Figures S11C‐F, Supporting Information). I–L) Zoomed‐in view of the boxed area in D–G). The scale bar denotes 50 µm. M) Calculated percentage of new bone from the SEM images D–G), and Figures S11C–F, Supporting Information). Non‐parametric Mann Whitney's test (*p < 0.05). Data are presented as mean ± standard error of the mean. Direct comparisons between FN‐ and NeoNectin‐grafted titanium implants were highlighted in red for both the post‐3‐week and 6‐week groups.

To evaluate the performance of the NeoNectin‐grafted biomaterials in vivo, we quantified the osseointegration of implants in a rabbit cortical bone model. In brief, two implants were inserted per tibia of a rabbit (**Figure** **6**A) so that each animal had all four conditions implanted (FN‐, NeoNectin‐, and RGD‐grafted, and bare titanium). Samples were retrieved 3 and 6 weeks after implantation and the quality and degree of osseointegration for each sample were evaluated. In all conditions during the study, we observed no signs of infection or inflammation in the animals.

First, we evaluated the amount of bone around the implant. Extensive bone matrix was observed in micro‐computerized tomography (micro‐CT) 3D reconstructions (Figure 6B; Figure S11A, Supporting Information). The average ratio of bone volume to total volume (BV/TV) was the highest for NeoNectin in both the 3‐week and 6‐week groups (45.3% and 55.0%, respectively, Figure 6C). In contrast, FN, RGD, and titanium groups showed significantly less bone ratio. In all conditions, we observed an increase in the BV/TV ratio from 3 weeks and 6 weeks after implantation.

Next, we evaluated the quality of the bone around the implants. The bone around the NeoNectin‐grafted implants appeared to be mostly compact and structured by histological staining and scanning electron microscopy (SEM) (Figure 6D–G; Figure S11D–G, Supporting Information). A more porous bone structure was noted at the interface of RGD‐grafted and bare titanium implants (Figure 6F,G; Figure S11F,G, Supporting Information), indicative of less mature bone. Furthermore, signs of fibrosis were mostly observed in RGD and bare titanium conditions by keratin red staining (Figure 6F,G; Figure S11F,G, left panels, Supporting Information). No signs of inflammation or infection were observed in the histological sections.

Then, we evaluated the bone integration by calculating the bone‐implant contact (BIC) from SEM images. In all conditions, the implants were integrated into the cortical bone after 3 weeks with similar BIC ratios (Figure 6D–G; Figure S11D–G, right panels, 6H, Supporting Information). However, all conditions except NeoNectin showed slightly decreased BIC values 6 weeks post‐implantation. The most plausible explanation for this overall decrease in BIC values is that the growing bone was not interacting properly with the implants. This is evident in high magnification SEM images where new bone appears dark gray, while old bone is light gray (Figure 6I–L). After quantification (see example in Figure S11B,C, Supporting Information), new bone around NeoNectin grafted implants showed the highest percentage relative to total bone at both 3 and 6 weeks of analysis (Figure 6M), suggesting that NeoNectin outperformed FN and RGD in promoting bone healing.

## 3 Discussion

Most current biomaterials used for tissue regeneration, such as titanium or 3D hydrogels, do not promote sufficient cell attachment for successful tissue integration in transplanted tissues. Hence, there is a growing need for finding biomolecules that can be used for coating and embedding biomaterials, making them more functional. Various levels of success have been achieved using RGD peptides or FN fragments, but their lack of specificity for various integrins and challenges in manufacturing constrains their application.Our 65‐amino acid designed NeoNectin, which exhibits hig affinity and specificity for integrin α5β1 and can be produced with high yield, shows considerable promise in overcoming these limitations. NeoNectin stablizes the EO conformation as does FN (Figure 3C; Figure S6J, Supporting Information).^[^ ^15^, ^16^ ^]^ While FN binds to both the α5‐β1 interface and a synergy site on α5, NeoNectin only binds to the interface between α5 and β1, suggesting that the synergy site binding is not essential for the conformational switching of integrin α5β1. Integrin α5β1 is crutial in early stages for recruiting MSCs in tissue regneration. A cyclic peptide specific for integrin α5β1^[^ ^21^, ^23^ ^]^ has also been shown to promote osteoblast adhesion, differentiation, and in vivo bone formation.^[^ ^42^, ^43^ ^]^

NeoNectin is a promising candidate for immobilization into any biomaterial for tissue regeneration—cells grown on NeoNectin‐grafted hydrogels or titanium implants completely spread and formed cytoskeletal fibers (Figure 5; Figure S9, Supporting Information). NeoNectin remained active upon implantation of grafted titanium, demonstrating the potential for use in bone integration or other implantation applications (Figure 6; Figure S11, Supporting Information). In vivo, NeoNectin significantly outperformed FN in promoting bone volume, enhancing bone‐implant contact, and generating more new bone, without evident off‐target effects. There were no signs of fever in rabbits implanted with either NeoNectin‐grafted or bare titanium implants, no inflammation or immune cell infiltration around the NeoNectin‐grafted material, no foreign body response or granuloma formation(Figure 6D–G; Figure S11D–G, Supporting Information) and no cytotoxicity was observed in MSCs, fibroblasts, osteoblasts, and HUVECs. *In‐silico* prediction using bioinformatics tools has shown no potential T‐cell/B‐cell epitopes (IEDB and NetMHC search), no allergenic potential (AllerTOP), and no toxic motifs (ToxinPred), supporting the safety profile of NeoNectin.^[^ ^46^, ^47^, ^48^, ^49^ ^]^

Collectively, these results showcase the possibility of using computationally‐designed proteins for regenerative medicine. Given the growing needs in this field, for example encapsulating therapeutic cells and/or recruiting/differentiating distinct cells during tissue healing, our approach of grafting materials with small, stable, and target‐specific designed proteins could be broadly applicable in regenerative medicine.

## 4 Experimental Section

### Resource Availability:Lead Contact

Further information and requests for resources and reagents should be directed to and will be fulfilled by the lead contact, David Baker (dabaker@uw.edu).

### Resource Availability:Materials Availability

This study did not generate new unique reagents.

### Resource Availability:Data and Code Availability

- RNA‐seq data have been deposited at GEO(GSE272058) and will be publicly available on the date of publication. All the data reported in this paper will be shared by the lead contact upon request.
- All original code has been deposited at https://github.com/xinruwang7/NeoNectin and is publicly available as of the date of publication.
- Any additional information required to reanalyze the data reported in this paper is available from the lead contact upon request.
- The coordinates of the atomic models have been deposited in the Protein Data Bank under accession codes 9CKV (open integrin with NN‐C1), 9DIA (closed integrin with NN‐C2), and 9EF2 (open integrin with NN‐C2).

### Experimental Model and Study Participant Details:Animals

A total of 20 mature New Zealand rabbit males were used in this study, each animal weighing 3.5–4 kg. All rabbits used were at 21–22 weeks of age. Ethical approval was obtained from the Ethics Committee of Consejería de Agricultura, Ganadería y Desarrollo Sostenible of the Junta de Extremadura (Spain) with ES 100 370 001 499 authorization code (December 20, 2023). The rabbits underwent general anesthesia for the surgical procedures using an intramuscular mixture of dexmedetomidine (Dexdomitor; Ecuphar, Barcelona, Spain), ketamine (Ketamidor; Karizoo, Barcelona, Spain) and buprenorphine (Bupaq; Richter Farma, Wells, Austria). Anesthetic maintenance was performed by inhalation with isoflurane (Isoflo, Zoetis, Madrid, Spain) at a fixed concentration of 1–2%. In addition, Lidocaine at 20 mg mL^−1^ (Braun, Kronberg im Taunus, Germany) was administered via an infiltrative route in the operated area. A single incision was made on the internal region of each tibia in all animals. A full‐thickness flap was opened, and randomized two implants were placed in the medial portion of each tibia near the epiphysis. Hence, four conditions were implanted in each animal. The implants were inserted according to the full‐drilling protocol, with bicortical anchorage, and separated by 6 mm in each tibia. A flat suture was made on the skin with simple stitches using 90% glycolide and 10% L‐lactide 4/0 resorbable suture (Vicryl 4/0 Ethicon, Johnson & Johnson International, USA) to facilitate adequate primary wound closure.

### Method details:Computational Design of Integrin α5β1 Binders

The ferredoxin scaffolds were generated in a piece‐wise assembly manner. The backbone information was first extracted for all existing ferredoxin‐like proteins reported by the SCOP database to generate guided blueprints for de novo ferredoxin. For the native ferredoxin‐like fold with secondary structure “EHEEHE,” there existed four beta‐sheets (E1, E2, E3, E4) and two helices (H1, H2) as basic elements, together with five loops (L1, L2, L3, L4, L5) connecting them in order. There existed optimal lengths and relative orientations between secondary structure elements. There also existed preferred torsion angles of the loops, which further used abego to determine these loop torsion patterns. Five most occurring abego were identified for each loop, 4 most occurring lengths for two helices (H1, H2), and 5 most occurring combinations of beta‐sheets. Combining all possible variables, the top 100 blueprints encoding ferredoxin topology information for the next step was finalized. Based on the blueprints generated, it was then applied the Rosetta blueprinter to create backbones constrained by the blueprints. To improve the success rate, each topology was built with 3 steps instead of constructing the whole protein in 1 step. Ferredoxin scaffold proteins were designed either starting with the RGD peptide (Fibronectin^1492‐1497^: 4WK2) or ferredoxin scaffold library used for RGD‐motif grafting in a later step. In the first case step 1, a helix and a beta strand were first built around the RGD peptide (E1+RGD+H1) with blueprint builder. After filtering with the Rosetta metrics, the top fragments were selected for step 2. In step 2, a beta‐hairpin at the C‐terminus of the H1 and parallel to the E1 (E1+RGD+H1+L2+E2+L3+E3) was further elongated and filtered the outputs with Rosetta monomer metrics. In step 3, the last L4+H2+L5+E4 fragments were built to make the whole ferredoxin. In the end, another round of filtering was imposed to remove designs with cavities and bad compactness. The trajectories that created the best‐designed scaffolds were further resampled to increase the number of designs. The filtered backbones generated from the last step were sequence‐optimized using Rosetta FastDesign and their structures were further evaluated via DeepAccNet^[^ ^14^ ^]^ or AlphaFold2.^[^ ^15^ ^]^ Designs with plddt <0.85 were dropped. In the second case, the ferredoxin scaffold libraries with altered steps were built.

### Integrin α5β1 Protein Purification and Biotinylation for Yeast Display

The integrin α5 (ITGA5, gene ID3678) and β1 (ITGB1, gene ID3688) ectodomain sequences were amplified by PCR reactions and inserted into the pD2529 vector. Integrin ectodomain was produced by co‐transfecting α and β subunit cDNAs with C‐terminal coiled coils^[^ ^50^ ^]^ into Expi293F cells using FectoPro (Polyplus) according to the manufacturer's instructions. The construct for the α5 subunit ectodomain in PD2529 CAG vector (ATUM) contains a N‐terminal CD33 secretion peptide (MPLLLLLPLLWAGALA) and C‐terminal HRV3C cleavage site (LEVLFQG), acid coil (AQCEKELQALEKENAQLEWELQALEKELAQ), Protein C tag (EDQVDPRLIDGK), and Strep twin tag (SAWSHPQFEKGGGSGGGGGSAWSHPQFEK). Construct for the β1 subunit ectodomain in PD2529 CAG vector contains an N‐terminal CD33 secretion peptide and C‐terminal HRV3C cleavage site, basic coil (AQCKKKLQALKKKNAQLKWKLQALKKKLAQ), HA tag (YPYDVPDYA), deca‐histidine tag, P2A (ATNFSLLKQAGDVEENPGP), and mCherry. 24 h of transfection, 3 mm valproic acid, and 4 g L^−1^ of glucose were added. After 7 days of transfection, the integrin ectodomain was purified from the culture supernatant using His‐Tag purification resin (Roche, cOmpelte, Cat No.5893682001), followed by size‐exclusion chromatography (GE Healthcare, AKTA purifier, Superdex 200) as clasped ectodomain form. The purified protein was biotinylated with EZ‐linkTM NHS‐Biotin (catlog 20 217, thermofisher) in 20 mm HEPES, pH 7.4, 150 mm NaCl, 1 mm Ca^2+^, 1 mm Mg^2+^, with 10 µm α5β1 ectodomain and 100 µm protein and EZ‐linkTM NHS‐Biotin, at 37 degrees Celsius for 16 h. Biotinylated α5β1 ectodomain was further purified by Superdex 200.

### Integrin α5β1 Protein Expression and Purification for BLI and Structural Determination

The integrin α5 (ITGA5, gene ID3678) and β1 (ITGB1, gene ID3688) ectodomain sequences were expressed in the pcDNA3.1‐Hygro(‐)‐TET vector. The construct for the α5 subunit ectodomain contains a C‐terminal HRV3C cleavage site (LEVLFQG), acid coil (AQCEKELQALEKENAQLEWELQALEKELAQ), and Strep‐tag (WSHPQFEK). The construct for the β1 subunit ectodomain contains a C‐terminal HRV3C cleavage site, base coil (AQCKKKLQALKKKNAQLKWKLQALKKKLAQ), and 6xHis tag. Integrin ectodomain was produced by co‐transfecting α and β subunit plasmids containing C‐terminal coiled coils^[^ ^50^ ^]^ into ExpiCHO cells (ThermoFisher) according to the manufacturer's instructions. The integrin ectodomain was purified from the culture supernatant using a HisTrap Prepacked Column (Cytiva), followed by overnight protease cleavage and size‐exclusion chromatography (GE Healthcare, AKTA purifier, Superdex 200).

### Yeast Surface Display Screening with FACS

The yeast surface display screening was performed using the protocol as previously described.^[^ ^14^, ^16^ ^]^ Briefly, DNAs encoding the minbinder sequences were transformed into EBY‐100 yeast strain. The yeast cells were grown in CTUG medium and induced in SGCAA medium. After washing with integrin‐FACS‐buffer (20 mm Tris, 150 mm NaCl, 1 mm Ca^2+^, 1 mm Mg^2+^, and 1% BSA), the cells were incubated with 1 um biotinylated target proteins (integrin ectodomains) together with streptavidin‐phycoerythrin (SAPE, ThermoFisher, 1:100) and anti‐c‐Myc fluorescein isothiocyanate (FITC, Miltenyi Biotech, 6.8:100) for 60 min. After washing twice with integrin‐FACS buffer, the yeast cells were then resuspended in the buffer and screened via FACS. Only cells with PE and FITC double‐positive signals were sorted for next‐round screening. After another round of enrichment, the cells were titrated with biotinylated target protein at different concentrations for 60 min, washed, and further stained with both streptavidin‐phycoerythrin (SAPE, ThermoFisher) and anti‐c‐Myc fluorescein isothiocyanate (FITC, Miltenyi Biotech) at 1:100 ratio for 30 min. After washing twice with integrin‐FACS buffer, the yeast cells at different concentrations were sorted individually via FACS and regrown for 2 days. Next, the cells from each subpool were lysated and their sequences were determined by next‐generation sequencing.

### Protein Binder Expression and Purification

Synthetic genes encoding designed proteins were purchased from Genscript or Integrated DNA Technologies (IDT) in the pET29b expression vector or as eBlocks (IDT) and cloned into customized expression vectors^[^ ^51^ ^]^ using Golden Gate cloning. A 6xHis tag was included either at the N‐terminus or the C‐terminus as part of the expression vector. Proteins were expressed using autoinducing TBII media (Mpbio) supplemented with 50 × 5052, 20 mm MgSO_4_, and Studier trace metal mix in BL21 DE3 *E.coli* cells (NEB: C2527H). Proteins were expressed under antibiotic selection at 25 ºC overnight after initial growth for 6–8 h at 37 ºC. Cells were harvested by centrifugation at 4000× g and resuspended in lysis buffer (20 mm Tris, 300 mm NaCl, 5 mm imidazole, pH 8.0) containing protease inhibitors (Thermo Scientific) and Bovine pancreas DNaseI (Sigma‐Aldrich) before lysis by sonication. One millimolar of the reducing agent TCEP was included in the lysis buffer for designs with free cysteines. Proteins were purified by Immobilized Metal Affinity Chromatography (IMAC). Cleared lysates were incubated with 0.1–2 mL nickel NTA beads (Qiagen) for 20–40 min before washing beads with 5–10 column volumes of lysis buffer, 5–10 column volumes of wash buffer (20 mm Tris, 300 mm NaCl, 30 mm imidazole, pH 8.0). Proteins were eluted with 1–4 mL of elution buffer (20 mm Tris, 300 mm NaCl, 300 mm imidazole, pH 8.0). All protein preparations were as a final step polished using size exclusion chromatography (SEC) on either Superdex 200 Increase 10/300GL or Superdex 75 Increase 10/300GL columns (Cytiva) using 20 mm Tris, 150 mm NaCl, pH 8.0. The reducing agent TCEP was included (0.5 mm final concentration) for designs with free cysteines. SDS‐PAGE and LC/MS were used to verify peak fractions. Proteins were concentrated to concentrations between 0.5–10 mg mL^−1^ and stored at room temperature or flash‐frozen in liquid nitrogen for storage at −80 ºC. Thawing of flash‐frozen aliquots was done at room temperature. All purification steps from IMAC were performed at room temperature.

### Enzymatic Biotinylation of Protein Binders

Proteins with Avi‐tags (GLNDIFEAQKIEWHE) were purified as described above and biotinylated in vitro using the BirA500 (Avidity, LLC) biotinylation kit. 840 µL of protein from an IMAC elution were biotinylated in a 1200 µL (final volume) reaction according to the manufacturer's instructions. Biotinylation reactions were allowed to proceed at either 4 °C overnight or for 2–3 h at room temperature on a rotating platform. Biotinylated proteins were purified using SEC on a Superdex 200 10/300 Increase GL (GE Healthcare) or S75 10/300 Increase GL (GE Healthcare) using SEC buffer (20 mm Tris pH 8.0, 100 mm NaCl).

### Peptide Synthesis

Peptides were synthesized on a 0.1 mmol scale via microwave‐assisted solid‐phase peptide synthesis (SPPS) LibertyBlue system (CEM) using preloaded Wang resin (CEM). The resin was subsequently treated with a cleavage cocktail consisting of TFA/TIPS/H2O/2,2‐(ethylenedioxy)diethanethiol in 92.5/2.5/2.5/2.5 proportions for 3 h, then precipitated in ice‐cold ether and washed twice before drying under nitrogen. The resulting crude was resuspended in water and a minimal amount of acetonitrile and purified on a semi‐preparative HPLC system (Agilent 1260 Infinity) with a linear gradient from solvent A to B of 2%/min (A: H2O with 0.1% TFA, B: acetonitrile (ACN) with 0.1% TFA). The peptide mass was confirmed via LC/MS‐TOF (Agilent G6230B) and lyophilized to a white powder. For grafting titanium discs and implants, a long RGD peptide was synthesized in order to ensure accessibility to cells. This long RGD peptide consists of a 3‐mercaptopropionic acid as anchoring moiety, 3 units of 6‐aminohexanoic acid as spacer, and the GRGDS sequence (MPA‐(Ahx)_3_‐GRGDS).

### Circular Dichroism Spectroscopy

CD spectra were recorded in a 1 mm path length cuvette at a protein concentration between 0.3–0.5 mg mL^−1^ on a J‐1500 instrument (Jasco). For temperature melts, data were recorded at 222 nm between 4 and 94 °C every 2 °C, and wavelength scans between 190 and 260 nm at 10 °C intervals starting from 4 °C. Experiments were performed in 20 mm Tris pH 8.0, 20 mm NaCl. The high tension (HT) voltage was monitored according to the manufacturer's recommendation to ensure optimal signal‐to‐noise ratio for the wavelengths of interest.

### Biolayer Interferometry (BLI)

The BLI experiments were performed on an OctetRED96 BLI system (ForteBio) at room temperature in integrin resting buffer (20 mm Tris pH 7.4, 150 mm NaCl, 1 mm MgCl_2_, 1 mm CaCl_2_, 0.02% Tween‐20) or active buffer (20 mm Tris pH 7.4, 150 mm NaCl, 1 mm MnCl_2_, 0.02% Tween‐20) or inactive buffer (20 mm Tris pH 7.4, 150 mm NaCl, 5 mm CaCl2, 0.02% Tween‐20) or low‐pH buffer (20 mm Tris pH 5, 150 mm NaCl, 1 mm MgCl_2_, 1 mm CaCl_2_, 0.02% Tween‐20). Each BLI buffer was supplemented with 0.2 mg mL^−1^ bovine serum albumin (Sigma‐Aldrich). Prior to measurements, streptavidin‐coated biosensors were first equilibrated for at least 10 min in the assay buffer. Protein binders with N‐terminal biotin were immobilized onto the biosensors by dipping them into a solution with 100 nm protein until the response reached between 10% and 50% of the maximum value followed by dipping sensors into fresh buffer to establish a baseline for 120 s. Titration experiments were performed at 25 ºC while rotating at 1000 rpm. Association of integrins was allowed by dipping biosensors in solutions containing designed protein diluted in octet buffer until equilibrium was approached followed by dissociation by dipping the biosensors into fresh buffer solution to monitor the dissociation kinetics. In the binding cross specificity assays each biotinylated binder was loaded onto streptavidin biosensors in equal amounts followed by 2 min of baseline equilibration. The association and dissociation with all the different binders were allowed for 900–3600 s for each step. Global kinetic or steady‐state fits were performed on buffer‐subtracted data using the manufacturer's software (Data Analysis 12.1) assuming a 1:1 binding model.

### Fluorescence Polarization

Binding affinity (or EC50) of the α5β1 binder to the soluble ectodomains of RGD‐binding integrins was analyzed by fluorescent polarization competitive binding assays. Affinities were measured by competing 10 nm FITC‐cyclic‐ACRGDGWCG (FITC labeled aminocaproic acid‐disulfide‐cyclized ACRGDGWCG peptide) binding to 200 nm αvβ1, 50 nm αvβ3, 50 nm αvβ5, 100 nm α5β1 or 1000 nm α8β1; 10 nm FITC‐proTGFβ3 peptide (FITC labeled aminocaproic acid‐GRGDLGRLKK peptide) binding to 10 nm αvβ6 or 250nm αvβ8. In the assay, 10 µL of sample contains the 10 nm FITC‐cRGD or FITC‐proTGFβ3, integrin ectodomain, and binder with indicated concentrations were incubated at room temperature for 2 h in the dark to ensure equilibrium before measurement. The buffer condition used for the reaction was 10 nm HEPES pH 7.5, 150 nm NaCl, 1 nm MgCl_2_, 1 nm CaCl_2_, and 0.5 mg mL^−1^ BSA. The competitive binding curves with binder or control as titrators on each individual integrin ectodomain were globally fitted with competitive binding equations,^[^ ^10^ ^]^ with the maximum FP value in the absence of titrator and the minimum FP value when all the integrin in solution being bound by titrators as global fitting parameters, and K_D_ value for each titrator as individual fitting parameter. When K_D_ cannot be reliably fitted, the EC50 was calculated by fitting the curve with a three‐parameter dose‐response curve using Prism (GraphPad Software, version 9). The errors are the standard errors from the nonlinear least square fits.

### Negative‐Stain EM Sample Preparation

The integrin‐binder complexes were formed using a 1:2 integrin to NeoNectin molar ratio, incubated at room temperature for at least 10 min, and diluted to a final concentration of 90 µg mL^−1^ (α5β1) in 20 nm Tris‐HCl pH 7.4, 150 nm NaCl, supplemented with either 1 nm MnCl_2_ or 5 nm CaCl_2_. 3 µL of the sample was applied to a glow‐discharged 400 mesh copper glider grid that had been covered with a thin layer of continuous amorphous carbon. The grids were stained with a solution containing 2% (w/v) uranyl format as previously described.^[^ ^39^ ^]^

### Negative‐Stain EM Data Acquisition and Processing

Data were acquired using a Thermo Fisher Scientific Talos L120C transmission electron microscope operating at 200 kV and recorded on a 4k × 4k Thermo Fisher Scientific Ceta camera at a nominal magnification of 92000× with a pixel size of 0.158 nm. Leginon^[^ ^52^ ^]^ was used to collect micrographs at a nominal range of 1.8–2.2 µm under focus and a dose of ≈50 e^−^ Å^−1^.^[^ ^2^ ^]^ Data sets collected in activating buffer conditions containing 1 nm MnCl_2_ had the following number of micrographs: 264 micrographs α5β1 alone, 331 micrographs α5β1 + NeoNectin, 335 micrographs α5β1 + NeoNectin candidate 2, 319 micrographs α5β1 + NeoNectin H34G/K35G, 474 micrographs α5β1 + NeoNectin W60A. Data sets collected in non‐activating buffer conditions containing 5 nm CaCl_2_ had the following number of micrographs: 329 micrographs α5β1 alone, 226 micrographs α5β1+ NeoNectin, 301 micrographs α5β1 + NeoNectin candidate 2, 484 micrographs α5β1 + NeoNectin H34G/K35G. Data were processed using Gautomatch (https://github.com/JackZhang‐Lab), RELION, and cryoSPARC.^[^ ^40^, ^41^, ^42^ ^]^

### Cryo‐EM Sample Preparation

The integrin binder complexes were incubated at room temperature for 30 min using a 1:2 integrin to binder molar ratio. From there, complexes were diluted to a final concentration of 90 µg mL^−1^ (α5β1) in 20 nm Tris pH 7.4, 150 nm NaCl, 1 nm MnCl_2_. For cryo‐EM grid preparation, UltrAufoil grids (300 mesh, 1.2/1.3) were glow‐discharged for 30 s at 15 mA, and then 3 µL of each complex were applied to each grid. Complexes were frozen with a Thermo Fisher Scientific Vitrobot Mark IV in 100% humidity at 4 °C and vitrified in liquid ethane cooled by liquid nitrogen.

### Cryo‐EM Data Acquisition and Processing

Datasets for 2 complexes were acquired on a Thermo Fisher Scientific Glacios cryo‐transmission electron microscope operating at 200 kV and recorded with a Gatan K3 Direct Detection Camera. For the NN‐C1 complex, a total of three datasets were collected from three separate grids. For the NN‐C2 complex, a single dataset was collected from a single grid. For data collection, the stage was tilted to 30° and all images were recorded using SerialEM software.^[^ ^53^ ^]^ One hundred frame movies were recorded in super‐resolution mode with a super‐resolution pixel size of 0.561 Å/px, a nominal magnification of 36kx, a nominal defocus range of 1.2 to 2.0 µm under focus, and an approximate dose of 50 e^−^ Å^−1^.^[^ ^2^ ^]^ For the NN‐C1 complex, 861 micrographs were used for subsequent data analysis (334, 183, and 204 micrographs from the respective data collections). For the NN‐C2 complex, 1231 micrographs were used. Dose fractionated super‐resolution image stacks were motion‐corrected and binned 2 × 2 using Fourier cropping with MotionCor2 within the RELION wrapper.^[^ ^54^ ^]^ Motion‐corrected stacks were processed using Patch CTF in cryoSPARC. For the NN‐C1 complex, 728379 particles were picked using the unbiased blob picker in cryoSPARC and subjected to iterative 2D and 3D alignment and classification yielding a final map at 3.28Å resolution (72604 particles). Additional local refinement of the binding interface gave a final map at an improved resolution of 3.19Å. For the NN‐C2 complex, 1150338 particles were picked using the unbiased blob picker in cryoSPARC and subjected to iterative 2D and 3D alignment and classification which resulted in two distinct headpiece conformations: closed and open. For the closed conformation, a map using 153759 particles was determined to a resolution of 2.98 Å. Additional local refinement improved details of the binding interface and had an overall resolution of 2.97 Å. For the open conformation, a map using 69.848 particles was determined to a resolution of 3.95 Å. Additional local refinement resulted in a map with a resolution of 3.56 Å. CryoEM processing details and results are summarized in Figure S4 (Supporting Information) (α5β1 + NeoNectin) and Figure S5 (Supporting Information) (α5β1 + NN‐C2).

### Model Building

The initial models for integrin model‐building were as follows: For the α5β1 + NN‐C1 complex, the open α5β1 headpiece structure (PDB: 7NWL) was used. For α5β1 + NN‐C2 complex, the closed α5β1 headpiece structure (PDB: 9B9J, submitted for publication) or the open α5β1 headpiece structure (PDB: 9CKV, this work) was used. For all model‐building, the predicted structure of the designed binder was used as an initial model. First, these models were fit into their respective cryo‐EM density using UCSF ChimeraX^[^ ^55^ ^]^ and glycans were manually added. Refinements were performed using COOT^[^ ^56^ ^]^ and ISOLDE.^[^ ^57^ ^]^ All maps (sharpened and unsharpened) used for modeling have been deposited. Cryo‐EM and model‐building statistics can be found in Table S1 (Supporting Information).

### Mammalian Cell Culture

Human umbilical vein endothelial cells (HUVECs) were purchased from Lonza (Catalog #: C2519AS) and cultured in EGM2 media as described previously.^[^ ^61^ ^]^ HUVECs were expanded and serially passaged to reach passage 4 before cryopreservation.

MCF10A cells were cultured in media as previously described;^[^ ^50^, ^51^ ^]^ briefly, the media consisted of DMEM/F12 (Gibco, 11 330 032), 5% horse serum (Gibco, 16 050 130), 20 ng mL^−1^ EGF (Sigma‐Aldrich, SRP3027), 0.5 mg mL^−1^ hydrocortisone (Sigma‐Aldrich, H4001), 100 ng mL^−1^ cholera toxin (Millipore, C8052), 10 µg mL^−1^ insulin (Sigma‐Aldrich,11070‐73‐8) and 1% penicillin–streptomycin (Gibco, 15 140 122). MCF10A cells were starved in the same media without EGF and contained 2% horse serum (assay media) for 16 h before signaling experiments.

Human bone marrow‐derived mesenchymal stem cells (MSCs; ATCC) and human osteoblasts (OBs; Sigma‐Aldrich) were cultured in Advanced DMEM medium supplemented with 10% FBS, 20 mm HEPES, penicillin/streptomycin (50 U mL^−1^ and 50 µg mL^−1^, respectively) and 2 mm L‐glutamine (all components from ThermoFisher Scientific). Cells from passage 5 were used in all experiments.

Human foreskin fibroblasts (FFs; Millipore), MG‐63 cells (ATCC), and A549 cells (Elabscience) were cultured in DMEM medium supplemented with 10% FBS, 20 mM HEPES, 50 U mL^−1^ penicillin, 50 µg mL^−1^ streptomycin and 2 mm L‐glutamine, all from ThermoFisher Scientific. FFs from passage 10 were used in all experiments.

SaOS‐2 cells (ATCC) were cultured in McCoy's 5A medium supplemented with 10% FBS, 20 mm HEPES, 50 U mL^−1^ penicillin, 50 µg mL^−1^ streptomycin and 2 mm L‐glutamine, all from ThermoFisher Scientific.

### Cell Binding Assays Using Flow Cytometry

CF647 Succinimidyl Ester (Biotium 92 135) was used to directly label NeoNectin following the manufacturer's protocol. To determine the affinity of CF647‐NeoNectin to α5β1 on the K562 cell surface (Figure 2J), 100 µL of cells (10^6^ mL^−1^) were mixed with indicated concentrations of CF647‐NeoNectin L15 medium containing 1% BSA for 2 h at room temperature and subjected to flow cytometry without washing. Background fluorescence was measured with 10 mm EDTA in the binding buffer. The background‐subtracted mean fluorescence intensity (MFI) at each concentration of CF647‐NeoNectin was fitted to a three‐parameter dose‐response curve for K_D_, background MFI, and maximum MFI.

Affinities of unlabeled NeoNectin, FN fragment (Fn3_9‐10_), and RGD peptide (GRRGDGATGH) for intact α5β1 on K562 cells were measured by competing CF647‐ NeoNectin binding (Figure 2K). Cells (10^6^ mL^−1^ in 100 µL) were mixed with 5 nm CF647‐NeoNectin and the indicated concentrations of competitor in L15 medium with 1% BSA. After 2 h in the dark at room temperature to ensure equilibrium, cells were subjected to flow cytometry without washing. MFI of CF647‐NeoNectin at each concentration of different competitors were globally fitted to three parameter dose‐response curves, with maximum MFI in absence of competitor and minimum background MFI as shared fitting parameters, and EC50 value for each competitor as individual fitting parameter. With the fitted EC50 value, K_D_ of each competitor was calculated as K_D_ = EC50 / (1+ C_L_/K_D, L_), where C_L_ is the concentration of CF647‐NeoNectin used (5 nm), and K_D, L_ is the binding affinity of CF647‐NeoNectin to α5β1 determined in Figure 2J.

### Covalently Grafting Titanium Surfaces with Different Biologics

NeoNectin and FN were covalently immobilized through their primary amine groups as previously described for other proteins.^[^ ^58^, ^59^ ^]^ Briefly, titanium discs of 10 mm diameter and 2 mm thickness were polished to remove the effect of roughness on cell attachment using silicon carbide papers and colloidal silica. Afterward, the discs were ultrasonically rinsed with cyclohexane, isopropanol, deionized water, ethanol, and acetone. Then, titanium discs were activated by oxygen plasma at 12 MHz in a Femto low‐pressure plasma system (Diener Electronic) and immersed in a 0.08 m solution of (3‐aminopropyl)triethoxysilane (APTES, Sigma‐Aldrich) at 70 °C for 1 h, rinsed with different solvents, and APTES was cross‐linked with 7.5 mm solution of N‐succinimidyl‐3‐maleimidopropionic acid. NeoNectin was then added at a 100 µg mL^−1^ (10 µm) concentration in PBS, being covalently linked through the amines of exposed lysines. FN was added at a 50 µg mL^−1^ concentration and covalently attached through its free amines. The RGD peptide (MPA‐(Ahx)_3_‐GRGDS) was chemically synthesized as described above and added at a 100 µm concentration in PBS at pH 6.5, and covalently attached through the free thiol. The surface chemical composition of grafted and bare titanium discs was analyzed using XPS. Spectra for C, O, N, Si, and Ti were acquired using an XR50 Al anode source operating at 150 W, coupled with a Phoibos 150 analyzer and an MCD‐9 detector on a SPECS Surface Nano Analysis system. High‐resolution spectra were recorded with a step size of 0.1 eV and a pass energy of 25 eV under ultra‐high vacuum conditions (7.5 × 10^−9^ mbar). Binding energies were referenced to the C 1s peak, calibrated at 284.4 eV. Coating thickness was estimated by the attenuation of the titanium signal, following established methodologies from previous studies.^[^ ^59^, ^60^ ^]^

### Cell Adhesion and Spreading Assay on Titanium Surface

MSCs, FFs, OBs, SaOS‐2, and MG‐63 were cultured in serum‐free conditions at a concentration of 25 000 cells per disc and allowed to adhere for 4 h (for vinculin staining and morphology evaluation) or for 24 h (for pFAK staining and blebbistatin treatment) on the grafted titanium discs. For blebbistatin treatment, cells were incubated for 1 h in a 20 µm solution after cells were attached for 24 h. Then, cells were fixed with 4% paraformaldehyde (PFA) for 20 min and rinsed with 20 mm glycine in PBS (washing buffer). Cells were permeabilized with 0.05% Triton X‐100 in PBS for 15 min, rinsed thrice with washing buffer, and blocked with 1% BSA for at least 30 min. Cells were then incubated with mouse anti‐vinculin (1:100; Sigma‐Aldrich) or anti‐pFAK (Tyr397; 1:100; Invitrogen) for 1 h, rinsed with washing buffer, and incubated with Alexa Fluor 488 goat anti‐mouse (1:1000; ThermoFisher Scientific) or Alexa Fluor 488 donkey anti‐rabbit (1:300; Invitrogen) and Alexa Fluor 546 phalloidin (1:300; ThermoFisher Scientific) for 1 h in the dark. Samples were mounted in a mounting medium containing DAPI for counterstaining the nuclei and visualized in an LSM 800 confocal microscope (Carl Zeiss). The area of cells and the circularity were calculated using the ImageJ software in images from at least five areas randomly selected.

### Tube Formation Assay

Tube formation assay was performed using previously described protocol.^[^ ^61^ ^]^ Briefly, passage 4 HUVECs were thawed onto a 10 cm 0.1% gelatin pre‐coated plate and cultured until 80–90% confluent. Before cell seeding, 150 µL of 100% matrigel was added to a pre‐chilled 24‐well plate to allow even spreading of the matrigel. The matrigel plate was allowed to solidify at room temperature for 25 min. HUVECs were seeded at 150 000 cells per 350 µL in each well with PBS or NeoNectin at 0.1 to 1000 nm, considering 500 µL as the total volume in each well. Cells were then imaged after 12 h. 20 images were taken in each well at random locations and images were analyzed using the Angiogenesis analyzer plugin in ImageJ. An average of the number of nodes, meshes, and segments of the 20 images, and these three parameters were also averaged to calculate the vascular stability for each well.

### Cell Adhesion Inhibition Assays

The glass bottom dishes (FluoroDish, FD35‐100, World Precision Instruments) were precoated with 50 µg mL^−1^ collagen‐I (Advance Biomatrix, #5056) or 5 µg mL^−1^ FN (Sigma, #F1141) for at least 3 h at 37 °C and washed with PBS. Integrin binder was added as indicated concentration on the precoated glass‐bottom dish for 30 min and cells were plated on these surfaces for an additional 30 min before fixing them with 4% PFA. The PFA fixed cells were permeabilized with the help of 0.1% Triton X‐100. Cells were blocked with 2% BSA, and 5% normal goat serum in PBS for 30 min followed by three washes with 1X PBS and incubated for 1 h with Alexa Fluor 647 Phalloidin (1:100 dilution) (Thermo, #A22287) for F‐actin staining. The images were taken after washing thrice with 1X PBS on an automated TIRF microscope (Nikon Ti, 100x/1.49 CFI Apo TIRF oil immersion objective) equipped with Perfect Focus, motorized x‐y stage, fast piezo z stage, and Andor iXon X3 EMCCD camera with 512×512‐pixel chip (16‐micron pixels). These images were processed and analyzed using ImageJ.

### Single‐Cell Migration Inhibition Assays

MCF10A cells (5 × 10^3^) were plated onto 12 wells plate in assay media. These cells were treated with the increasing concentration (0, 20, 200, 500 nm) of integrin mini binder (mb) after 12 h of attachment and imaged once every 10 min for 18 h on an IncuCyte S3. Images were processed using ImageJ software and analyzed using a manual tracking plugin.

### RNA‐Sequencing

To assess how soluble NeoNectin regulates gene expression by interfering with FN‐mediated adhesion, MCF10A cells were seeded onto FN‐coated plates in the presence or absence of soluble NeoNectin. MCF10A cells were harvested after 4 h of incubation for downstream gene expression analysis. MSCs and FFs were prepared as described in cell attachment and spreading experiments. 5 × 10^4^ –1 × 10^5^ cells were harvested. RNA was prepared by directly lysing cells in plates with 350 µL RLT Plus with B‐Me and processed with the RNAEasy Plus mini kit (Qiagen cat. no 74 134) to obtain gDNA‐eliminated total RNA. RNA was further processed into bulk RNA‐seq libraries (1 ug input per library) in duplicate with an Illumina Stranded mRNA prep kit (Illumina cat. no 20 040 532) according to the manufacturer's instructions. Final libraries were quantified and characterized with an Agilent High Sensitivity D1000 ScreenTape (Agilent cat. no 50 675 584). Libraries were sequenced on an Illumina P2 100 cycle kit with the following parameters: 10:59:59:10 (index1:read1:read2:index2). Data was demultiplexed with bcl2fastq and preprocessed with BioJupies. The resulting count matrices were log CPM normalized per sample and z‐scored across conditions to compare expression levels. Limma was used to compare DEGs. Enrichr was used for GSEAs.

### Hydrogel

The dicysteine crosslinking peptide Ac‐GCRDLPESGGPQGIWGQDRCG‐NH_2_ was purchased from Genscript (Piscataway, NJ). The FN‐derived adhesion sequence CRGDS was synthesized on rink amide ProTide resin (CEM Corporation; Charlotte, NC) following induction‐heating assisted Fmoc solid‐phase techniques with HCTU activation (Gyros Protein Technologies PurePep Chorus; Tucson, AZ) at a 0.2 mmol scale. The resin was treated with a trifluoroacetic acid (TFA)/ethane dithiol (EDT)/water/triisopropylsilane (94:2.5:2.5:1) mixture for 3 h, then precipitated and washed in ice‐cold diethyl ether (2 × 150 mL). The crude peptide was purified via semi‐preparative reversed‐phase high‐performance liquid chromatography with a linear gradient of 5–100% acetonitrile and 0.1% TFA for 45 min and then lyophilized to yield a white powder of the final peptide CRGDS. Peptide mass was verified via ESI‐LCMS. Both peptides were resuspended in 10% acetic acid and lyophilized to yield aliquots of the desired mass.

For MSC encapsulation, all gel precursors were combined at 3 mM 4‐arm Poly(ethylene glycol) norbornene terminated (PEG‐NB; JenKem): 12 mm dicysteine peptide: 1 mm lithium phenyl‐2,4,6‐trimethylbenzoylphosphinate (LAP; Allevi3D). RGD peptide (CRGDS) or the NeoNectin variants were included in the final formulation at a concentration of either 0.5 or 1 mm. MSCs were resuspended in the gel mixture at a concentration of 1 × 10^6^ cells mL^−1^ and 5 µL gels were pipetted on the bottom of a 96 well‐plate, at which point they were exposed to collimated near‐UV light (λ = 365 nm; 10 mW cm^−2^; Omnicure 1500) for 2 min to allow for thiol‐ene polymerization. The gels were then covered in media and cultured for 5 days. On day 5, gels were fixed by treatment with 4% PFA for 1 h at room temperature and then washed 3 × 10 min with PBS and permeabilized for 30 min with 0.5% Triton X‐100 in PBS. Subsequently, actin was labeled with 1:400 Phalloidin AF‐532, and nuclei–‐with 1:1000 Hoechst 33 342 in PBS. Gels were rinsed in PBS and imaged on a Leica Stellaris confocal microscope. Cell area and eccentricity were analyzed with Cell Profiler 4.0.^[^ ^62^ ^]^

### Colocalization Imaging

MCF10A cells were plated on 35 mm glass‐bottom dishes (FluoroDish, FD35‐100, World Precision Instruments) for 24 h. These cells were treated with C6‐GFP‐NN for 30 min and incubated at 37 °C containing 5% CO_2_. Cells were fixed with 4% PFA and permeabilized with 0.1% Triton X‐100. The non‐specific antigens were blocked with blocking reagents (2% BSA+3% normal goat serum), which was followed by incubation with primary antibodies for 2 h. The antibodies used in the study are rat anti‐integrin‐β1 (9EG7, 553 715) mouse anti‐Rab‐5 (BD Transduction Laboratories, 610 724), mouse anti‐Rab‐11 (BD Transduction Laboratories, 610 656), mouse anti‐EEA‐1 (BD Transduction Laboratories, 610 456). The cells were washed with 1X PBS thrice before adding appropriate secondary antibodies. These cells were mounted in ProLong Gold (Invitrogen) for confocal microscopy using a Dragonfly 200 High‐speed Spinning disk confocal imaging platform (Andor Technology Ltd) on a Leica DMi8 microscope stand equipped with a ×100/1.4 oil immersion objective, iXon EMCCD and sCMOS Zyla cameras and Fusion Version 2.3.0.36 (Oxford Instruments) software together with Imaris simultaneous deconvolution. These images were used to evaluate the percentage of colocalization using Mander's colocalization coefficient between Rab5 and integrin binder with ImageJ plugin Coloc 2.

### Animal Implantation

Titanium implants of 3 mm diameter and 8 mm length with a conventional sandblasted, large grit, acid‐etched (SLA) surface (Klockner Vega implants; Soadco, Andorra) were functionalized as described above for titanium discs. Four conditions were prepared: (i) FN, (ii) NeoNectin, (iii) MPA‐(Ahx)3‐GRGDS (RGD peptide) (iv) bare Ti. After 3 and 6 weeks, animals were euthanized (8 animals per time) using the same anesthesia protocol mentioned in the animals section followed by an injection of potassium chloride (1–2 mmol kg^−1^). The tibia bones were harvested and immersed in 10% formaldehyde solution for at least one week. Afterward, samples were dehydrated in increasing ethanol concentrations (50%, 70%, 100%) for at least 2 days in each solution.

### Micro‐Computerized Tomography (Micro‐CT) Analysis

Quantification of bone around the implants was performed using a SkyScan 1272 X‐ray Micro‐CT scanner (Bruker, USA). Images were acquired at every 0.3° and a resolution of 2016 × 1334 with a pixel size of 10 µm for a complete 360° rotation. Images were then analyzed using the CT‐Analyzer software (CTAn, Bruker). A volume of interest (VOI) was selected around the implants. The NRECON software (Bruker) was used to obtain 3D reconstruction images.

### Scanning Electron Microscopy (SEM)

Samples were immersed in ethanol solutions containing increasing concentrations (50%, 70%, 90%, 100%) of methyl‐methacrylate resin Technovit 7200 (Kulzer‐Heraus, Germany). Samples were then stored in vacuum for 24 h to ensure resin penetration into the tissues, and the resin was photopolymerized using a Histolux light control unit (Kulzer‐Heraus) for 24 h. The samples were cut in two halves perpendicular to the longitudinal axis of the bone to expose the metallic implants. One of the two halves was polished with 800, 1200, and 4000 SiC abrasive papers and gold‐coated by sputtering before visualization in a Phenom XL Desktop SEM. Images were acquired at a working distance of 4 mm and a voltage of 15 kV, and analyzed using the QuPath software.^[^ ^63^ ^]^ Bone‐to‐implant contact (BIC) was calculated using ImageJ as previously described elsewhere.^[^ ^64^ ^]^

High‐resolution images were acquired using a Neon40 Crossbeam FIB‐SEM (Carl Zeiss, Germany) at a voltage of 15 kV with a working distance of 8 mm. The percentage of new bone versus total bone was calculated using the QuPath software.

### Histological Staining

Samples were further cut into 500 µm sections with a diamond saw and afterward polished until 100 µm with SiC abrasive papers. The sections were then stained by Masson's trichrome staining. Briefly, sections were first stained in Weigert's hematoxylin for 15 min for staining nuclei and rinsed with tap water for 5 min. Thereafter, sections were stained with Goldner I solution for 7 min and phosphomolybdic acid for 5 min, both for staining connective tissue in red, rinsing with 2% acetic acid after each staining. Finally, sections were stained with Light Green SF solution for 15 min for staining bone in green and rinsed with 2% acetic acid. Sections were then rinsed in tap water and mounted for visualization using an LSM confocal laser scanning microscope (Carl Zeiss, Germany) in stitching mode.

### Visualization

Protein structures were analyzed and visualized using ChimeraX^[^ ^65^ ^]^ and Pymol.^[^ ^66^ ^]^ The cartoons shown in Figures 1, 4 and 6A were created with the assistance of BioRender.^[^ ^67^ ^]^

### Statistical Analysis

Data from in vitro experiments are presented as mean ± standard deviation. Each experiment was performed independently three times, with at least three technical replicates per condition unless otherwise specified. RNA‐sequencing data was collected with two technical replicates per condition. Statistical significance for volcano plots was determined by Benjami‐Hochberg adjusted p‐values (<0.05 as significant). Enrichr was used for GSEAs. Limma was used to compare DEGs. Statistical significance between groups (*p* < 0.05) was determined using one‐way ANOVA followed by Tukey's post‐hoc test.

Data from in vivo experiments are presented as mean ± standard error of the mean. These experiments were conducted using 16 animals, as described above. Statistical comparisons between groups (*p* < 0.05) were performed using the non‐parametric Mann‐Whitney test with Bonferroni correction for multiple comparisons. All statistical analyses were conducted using Minitab software.

## Conflict of Interest

X.W., J.G.‐M., B.H., and D.B. are co‐inventors on an International patent (Serial 63/570,567) filed by the University of Washington covering molecules and their uses described in this manuscript. A.R., X.W., and D.B. are co‐founders of Lila Biologics and own stock or stock options in the company.

## Author Contributions

X.W. and J.G.‐M. contributed equally to this work. X.W., J.G.‐M., S.K, D.L., and D.B. designed the research; X.W and J.G.‐M. designed the initial library; X.W performed the screening experiments and analysis for the initial library and affinity maturation; X.W. designed the MPNN‐version binder and added a disulfide bond to the designed mini binders; X.W. designed, cloned, expressed, and purified binder constructs for characterization of the binding affinity and specificity to integrin α5β1, cell assay; B.H. designed the ferredoxin scaffold library; J.G.‐M. prepared the covalently linked titanium chips and performed the cell adhesion and spreading assay on the titanium surface; J.G.‐M. performed real‐time PCR and quantified the integrins expressed in cells; S.K. performed the cell attachment and migration inhibition assay and colocalization of binder bound α5β1 with various cellular components; J.G.‐M. and S.K. harvested cells for RNA sequencing experiments; D.L. performed RNA extraction and bulk RNA sequencing and analysis; X.W. and K.A.E.A. performed BLI assays; X.W. and D.L. performed the cell binding assays; Y.L. performed the specificity assay using fluorescence polarization; Y.T.Z. performed the HUVEC tube formation assay; I.K. and C.A.D. designed and performed the hydrogel‐based cell encapsulation studies; K.A.E.A. and A.N. prepared integrin α5β1 used for EM and cryo‐EM studies; J.L. and K.A.E.A. prepared the integrin α5β1 used for yeast‐display assay and BLI; K.A.E.A. and R.W. prepared samples and collected data for EM and cryo‐EM; K.A.E.A., R.W., and M.G.C. processed the data and built the cryo‐EM model; X.W., R.W, K.A.E.A., A.N., and M.G.C. analyzed the EM and cryo‐EM models. A.B.‐V. performed rabbit surgical interventions; J.G.‐M. and D.C.‐A. prepared implant samples and processed the tissues for histomorphometric analyses; X.W. and J.G.‐M. wrote the initial manuscript. All authors contributed to the edition and discussion of the manuscript.

## Supporting information



Supporting Information

## Data Availability

The data that support the findings of this study are available in the supplementary material of this article.
