# Supplementary material for: De Novo Design of Integrin α5β1 Modulating Proteins to Enhance Biomaterial Properties
Source: Adv Mater. 2025 Jun 9;37(34):2500872. doi: 10.1002/adma.202500872 (PMC12392869; doi:10.1002/adma.202500872)
Supplement: Supplementary file 1 — Supporting Information [file ADMA-37-2500872-s001.docx]

**Supplemental items**

Figure S1. Experimental screening of α5β1 binders, related to Figure 1.

Figure S2. Competition curves, and calculated K_D_ values from fluorescence anisotropy, related to Figure 2.

Figure S3. Colocalization of NeoNectin with ITGB1 and endosomal markers in MCF10A cells, related to Figure 2.

Figure S4. Cryo-EM data processing schematic of NeoNectin + α5β1, related to Figure 3.

Figure S5. Cryo-EM data processing schematic of NeoNectin Candidate 2 + α5β1, related to

Figure S6. Structural analysis of integrin α5β1 in complex with NeoNectin candidates, cyclic RGD, and fibronectin (related to Figure 3).

Figure S7. Soluble NeoNectin inhibits α5β1-mediated cellular behaviors, related to Figure 4.

Figure S8. Hydrogel modification enhances cells spreading, related to Figure 5.

Figure S9. Modulation of cell adhesion by NeoNectin immobilized onto Ti discs, related to Figure 5.

Figure S10. Scatterplots of gene expression against bare Ti discs for FN-, NeoNectin-, and RGD-grafted Titanium discs, related to Figure 5.

Figure S11.: NeoNectin-grafted titanium implant outperforms FN- and RGD- grafted, and bare titanium implants in stimulating implant integration and bone growth, related to Figure 6.

Table S1. Cryo-EM Data Collection and Processing Statistics.

Table S2. Surface elemental atomic composition (%) and thickness (nm) of grafted titanium discs.

**
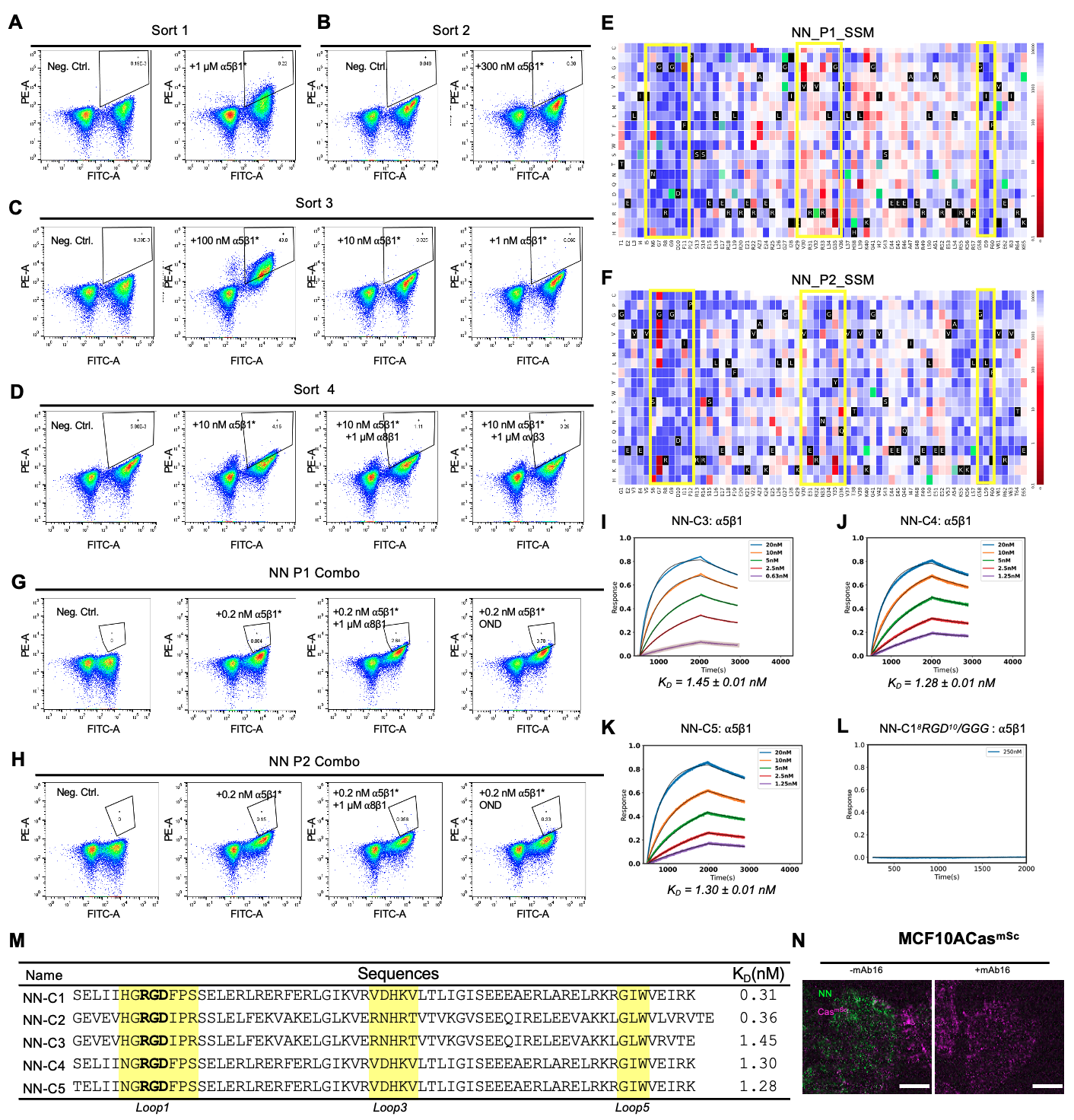
**

**Figure S1. Experimental screening of α5β1 binders, related to Figure 1**.

(A-D) Yeast cells displaying the SSM library of integrin α5β1 binders were incubated with various concentrations of biotinylated α5β1 in the presence or absence of non-labelled αvβ3 or α8β1. Labeled α5β1 binding to cells (y axis) was monitored with flow cytometry.

(E-F) SSM analysis of NN parent design 1 (NN P1)and parent design 2 (NN P2). Loop1, loop3 and loop5 were highlighted. The affinity of each variant was colored by red to white to blue gradient: red indicates the tightest binders, blue the weakest binders, and green indicates variants without sufficient data for analysis.

(G-H) Combinatorial libraries of the mutations that improved binding affinity, as identified by the red positions in Figure E and F.

(I-K) BLI binding affinity traces for NeoNectin candidates against α5β1 in the resting buffer (20 mM Tris, pH = 7.4, 1 mM Ca^2+^, 1 mM Mg^2+^). From left to right, K_D_= 0.4, 1.5, 1.3, and 1.3 nM respectively.

(L) BLI binding affinity traces for NeoNectin ^8^RGD^10^/GGG mutant against α5β1.

(M) Summary of NeoNectin candidate sequences and their binding affinities against α5β1 measured by BLI.

(**N**) TIRF images of MCF10A cells treated with/without 200 nM α5β1-specific antibody mAb16 followed by addition of 20 nM GFP-tagged NeoNectin. Green: GFP-tagged NeoNectin. Each dot (in magenta) represents an integrin-positive structure marked by the presence of Cas protein. The representative images are of a single-cell image of MCF10A cells taken at 100X. The scale bar is 10 µm.

**
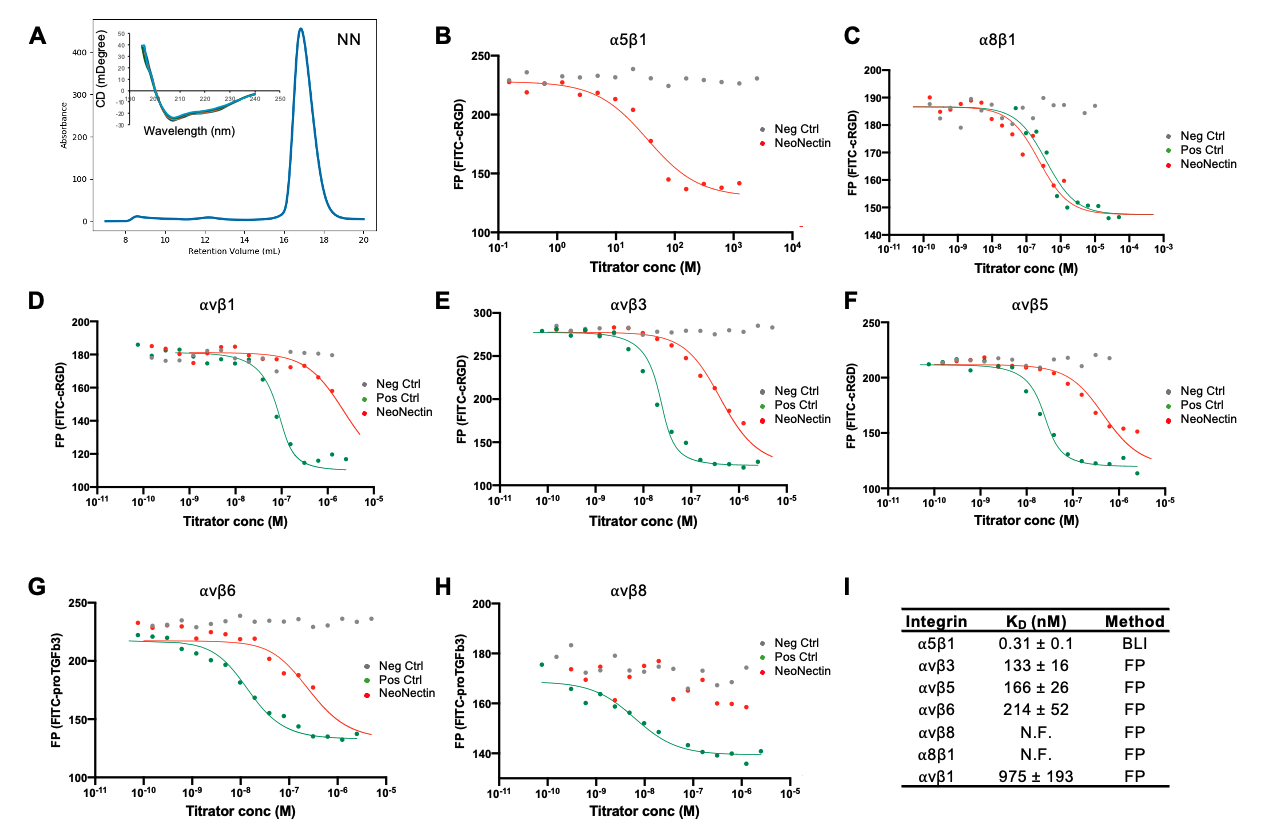
Figure S2. Competition curves, and calculated K_D_ values from fluorescence anisotropy, related to Figure 2.**

(A) Size Exclusion Chromatography trace of NeoNectin purified from 40 milliliter (ml) media and Circular dichroism spectra of NeoNectin at different temperatures (navy, 25 °C; orange, 55 °C; green, 75 °C, blue 95 °C).

(B) Binding affinity of NeoNectin to integrin α5β1 was measured by competing 10 nM FITC-cyclic-RGD binding to 100 nM α5β1. Neg Ctrl: αvβ3_ab7. K_D_ couldn’t be fitted.

(C) Binding affinity of NeoNectin to integrin α8β1 was measured by competing 10 nM FITC-cyclic-RGD binding to 1000 nM α8β1. Neg Ctrl: αvβ3_ab13; Pos Ctrl: cRGD

(D) Binding affinity of NeoNectin to integrin αvβ1 was measured by competing 10 nM FITC-cyclic-RGD binding to 200 nM αvβ1. Neg Ctrl: αvβ3_ab13; Pos Ctrl: αvβ1_ab5

(E) Binding affinity of NeoNectin to integrin αvβ3 was measured by competing 10 nM FITC-cyclic-RGD binding to 50 nM αvβ3. Neg Ctrl: αvβ6_ab4; Pos Ctrl: αvβ3_ab13

(F) Binding affinity of NeoNectin to integrin αvβ5 was measured by competing 10 nM FITC-cyclic-RGD binding to 50 nM αvβ5. Neg Ctrl: αvβ3_ab13; Pos Ctrl: αvβ5_ab9

(G) Binding affinity of NeoNectin to integrin αvβ6 was measured by competing 10 nM FFITC-proTGFβ3 binding to 10 nM αvβ6. Neg Ctrl:αvβ3_ab7; Pos Ctrl: αvβ6_ab6

(H) Binding affinity of NeoNectin to integrin αvβ8 was measured by competing 10 nM FFITC-proTGFβ3 binding to 250 nM αvβ8. Neg Ctrl:αvβ3_ab13 ; Pos Ctrl: αvβ8_ab4 (dual specific to αvβ6 and αvβ8).

(I) Binding affinity of NeoNectin to the soluble ectodomains of RGD-binding integrins by fluorescent polarization competitive binding assays from C to H.

**
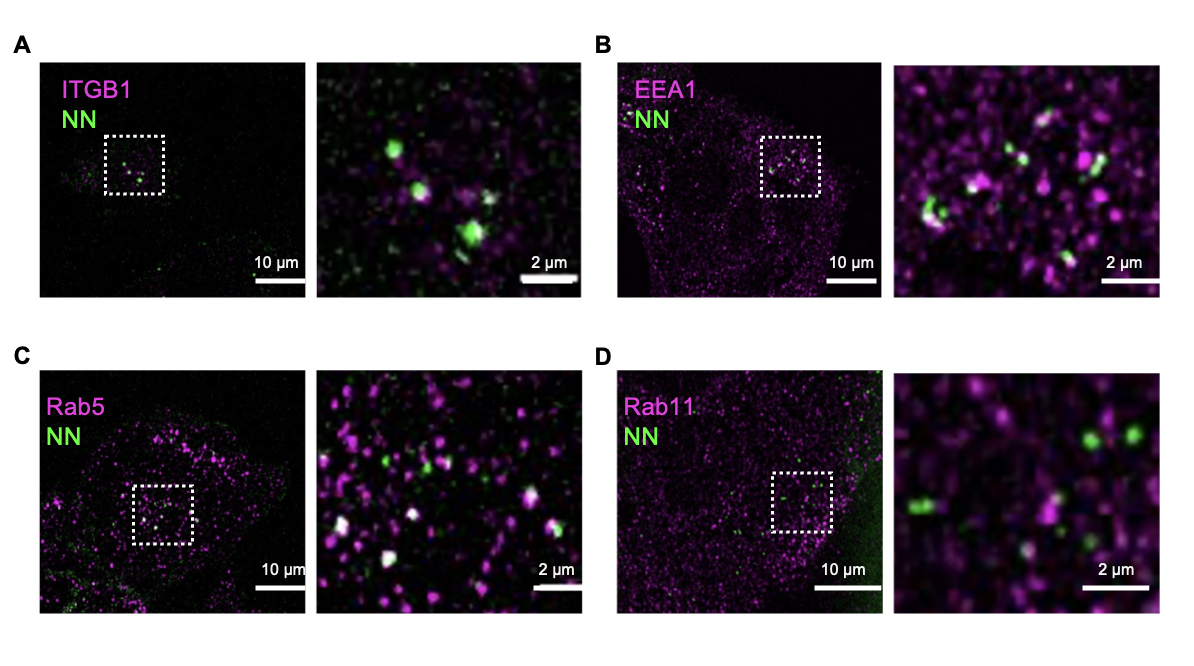
Figure S3. Colocalization of NeoNectin with ITGB1 and endosomal markers in MCF10A cells, related to Figure 2.**

(A-D) Cross-section confocal images showing colocalization of NeoNectin (GFP-tagged) and integrin β1 subunit (A), Early Endosome Antigen 1 (EEA1, B), Ras-related protein 5 **(**Rab5, C), and Rab11 (Ras-related protein Rab-11) . Right panel is a zoomed view of the left panel. Scale bars are 10 µm and 2 µm, respectively.

**
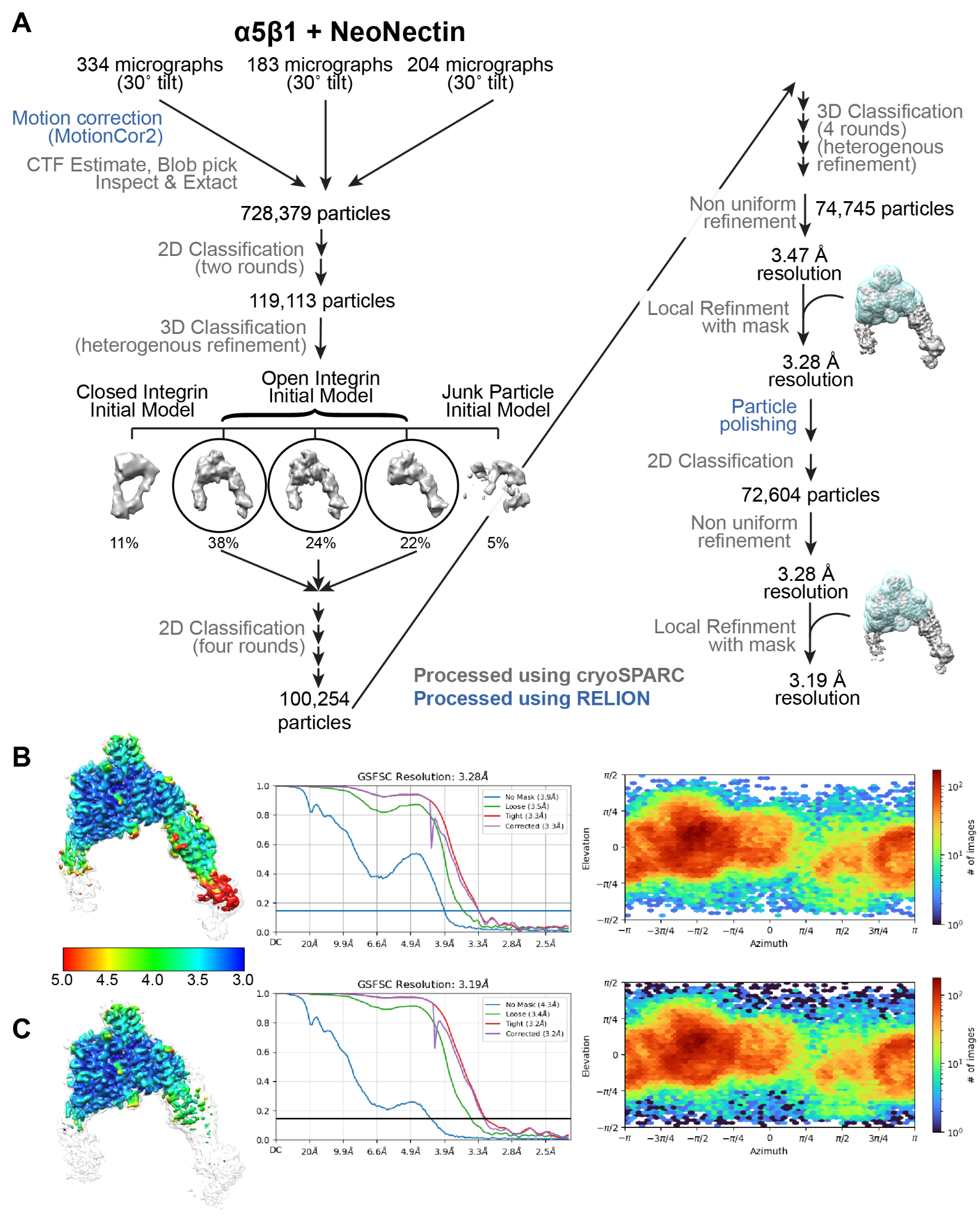
Figure S4. Cryo-EM data processing schematic of NeoNectin + α5β1, related to Figure 3.**

(A) An overview summarizing cryo-EM data processing for the α5β1+NeoNectin complex. Particle numbers at key steps and percentages for each class are indicated.

(B) Left: Sharpened map of α5β1+NeoNectin complex colored based on a local resolution, unsharpened map shown in semi-transparent white at a lower threshold. Middle: Gold-standard Fourier shell correlation plot. Right: Orientational distribution plot of the α5β1 in complex with NeoNectin.

(C) Left: Final local refined and sharpened map of α5β1+NeoNectin complex colored based on a local resolution, unsharpened map shown in semi-transparent white at a lower threshold. Middle: Gold-standard Fourier shell correlation plot. Right: Orientational distribution plot of the locally-refined α5β1 in complex with NeoNectin.


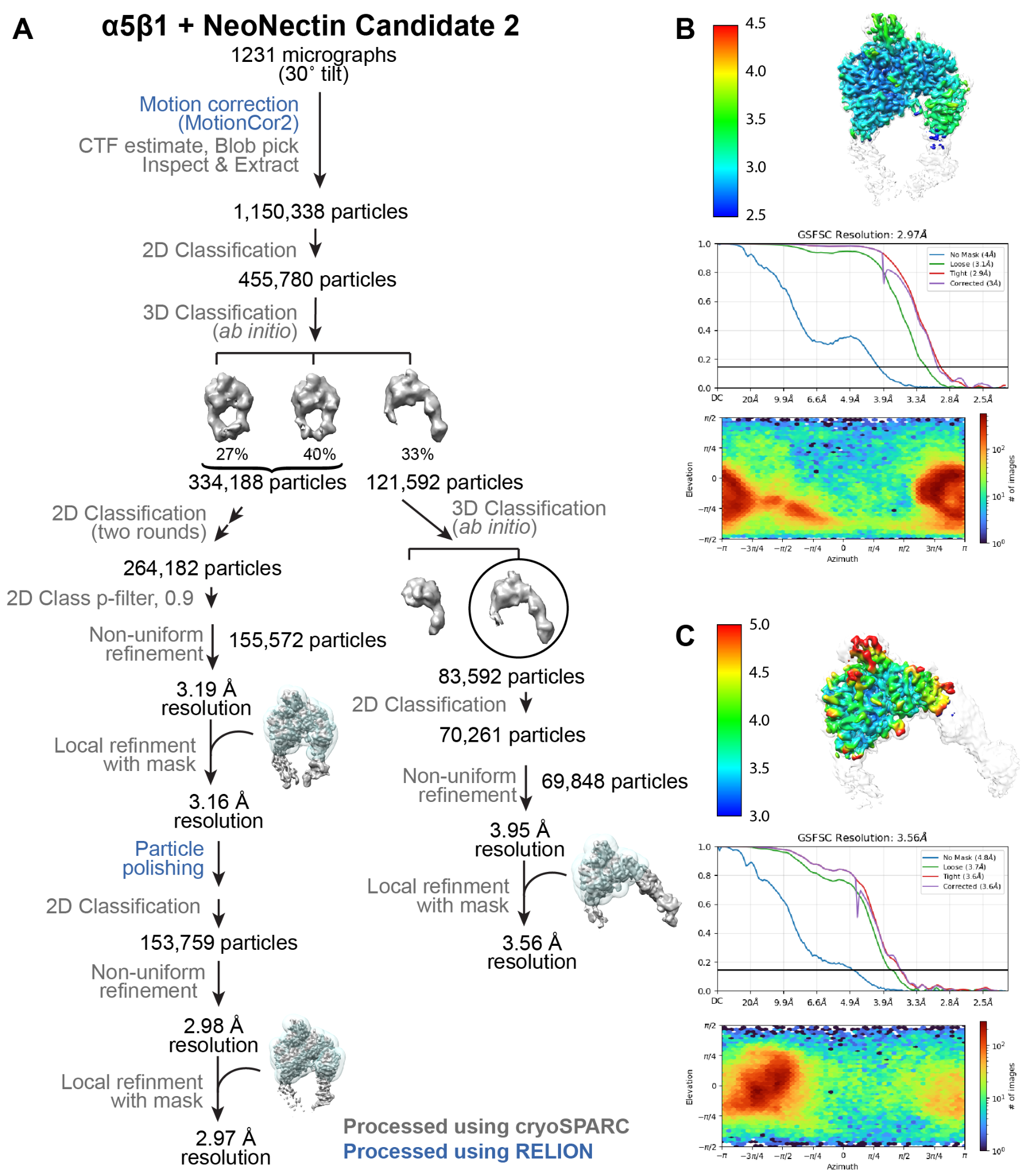
**Figure S5. Cryo-EM data processing schematic of NeoNectin Candidate 2 + α5β1, related to Figure 3**

(A) An overview summarizing cryo-EM data processing for the α5β1+NeoNectin Candidate 2 complex. Particle numbers at key steps and percentages for each class are indicated.

(B) Top: Sharpened map of Closed Headpiece α5β1+NeoNectinCandidate 2 complex colored based on a local resolution, unsharpened map shown in semi-transparent white at a lower threshold. Middle: Gold-standard Fourier shell correlation plot. Bottom: Orientational distribution plot.

(C) Top: Sharpened map of Open Headpiece α5β1+NeoNectinCandidate 2 complex colored based on a local resolution, unsharpened map shown in semi-transparent white at a lower threshold. Middle: Gold-standard Fourier shell correlation plot. Bottom: Orientational distribution plot.


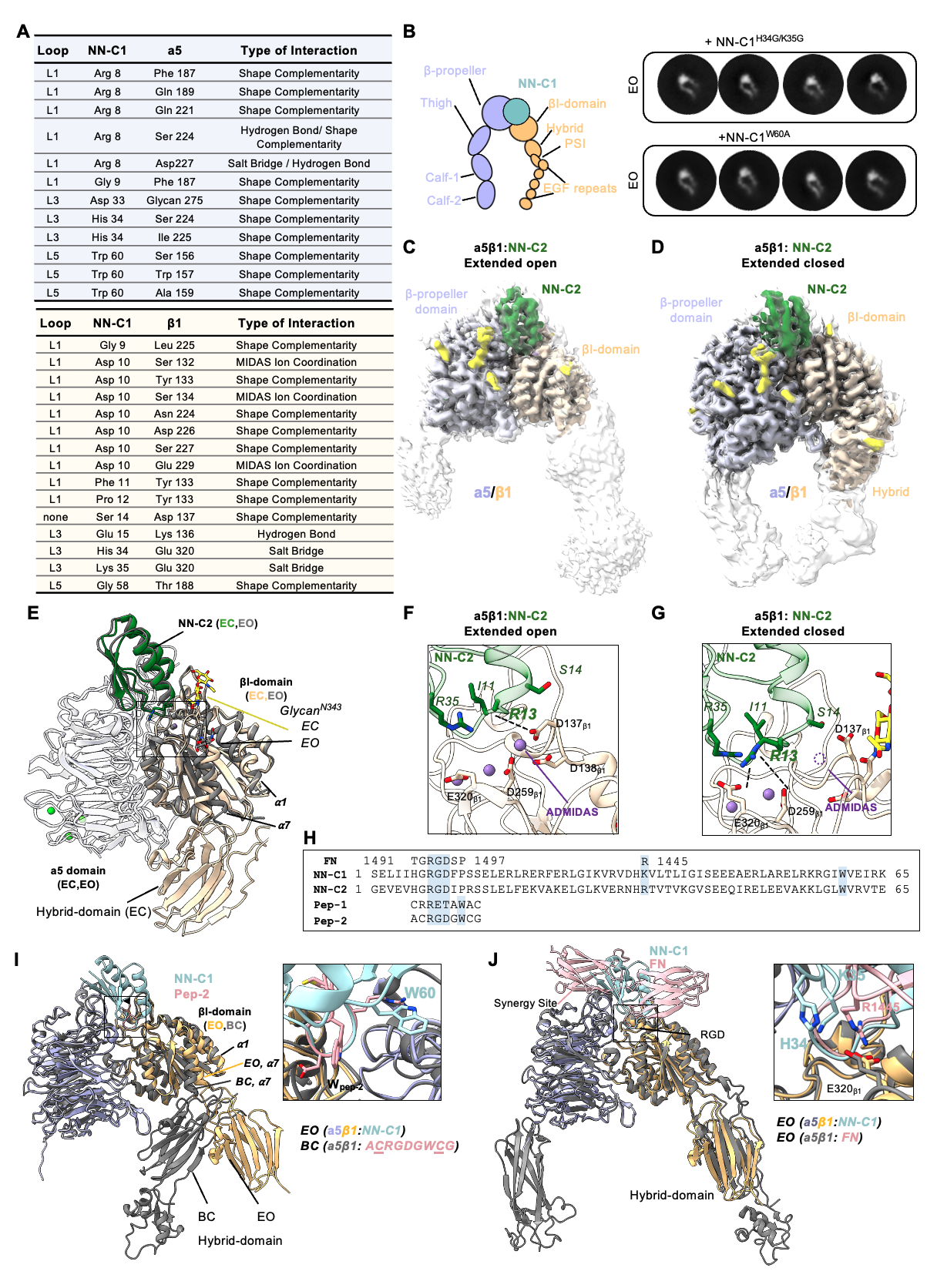


**Figure S6 Structural analysis of integrin α5β1 in complex with NeoNectin candidates, cyclic RGD, and fibronectin (related to Figure 3)**

(A) Summary of interactions between NN-C1 and α5β1.

(B) Left; cartoon model of an integrin receptor in extended open conformation; Right: Representative 2D negative stain class averages of α5β1 in presence of α5β1 NN-C1 variants in activating (1 mM Mn^2+^) buffer conditions. Integrins are categorized into the three canonical conformations: extended open (EO), extended closed (EC) and bent closed (BC).

(C, D) Cryo-EM map of α5β1 bound to NeoNectin candidate 2 (NN-C2) in extended open (F) or extended closed (G) state. The sharpened, locally refined map is shown in color, superimposed with the unsharpened map in semi-transparent white. The color code is as follows: α5 (lavender), β1 (light orange), Neonectin (forest green), coordinated cations (plum), glycans (yellow).

(E) Overlay of extended open (grey) and extended closed (colored) NN-C2 bound integrin α5β1 complexes. The Highlighted area is shown in detail in F and G.

(F) Close-up of extended open NN-C2:α5β1. Arg13_NN-C2_ forms a salt bridge with integrin β1 Asp137.

(G) Close-up of extended closed NN-C2:α5β1. Arg13_NN-C2_ forms salt bridges with integrin Asp259_β1_ and Glu320_β1_ . AMIDAS is absent in this EC conformation.

(H) Sequence comparison of NeoNectin variants, NN-C1 and NN-C2, with two previously reported cyclic peptides, specific for integrin α5β1. Pep-1: cyclic CRRETAWAC; Pep-2: cyclic ACRGDGWCG. Cyclization occurs through the underlined cysteine residues.

(I) Left: Overlay of cyclic-ACRGDGWCG-bound integrin α5β1 (PDB: 4WK4, bent closed, BC) and NN-C1-bound (extended open, EO) integrin α5β1 complexes; Right: Close-up view highlighting W60 of NN-C1 and the W in the ACRGDGWCG interface with α5β1. NN-C1-bound α5β1 is colored as follows: α5 (lavender), β1 (light orange), NeoNectin (turquoise). ACRGDGWCG-bound α5β1 is shown in grey with ACRGDGWCG in pink.

(J) Left: Overlay of fibronectin-bound integrin α5β1 (PDB: 7NWL,extended open, EO) and NN-C1-bound integrin α5β1 complexes (extended open, EO); Right: Close-up view highlighting H34,K35 of NN-C1 and the R1445 in the fibronectin interface with α5β1. Synergy Site and RGD loop are highlighted. NN-C1-bound α5β1 is colored as follows: α5 (lavender), β1 (light orange), NeoNectin (turquoise). Fibronectin-bound α5β1 is shown in grey with fibronectin in pink.

**
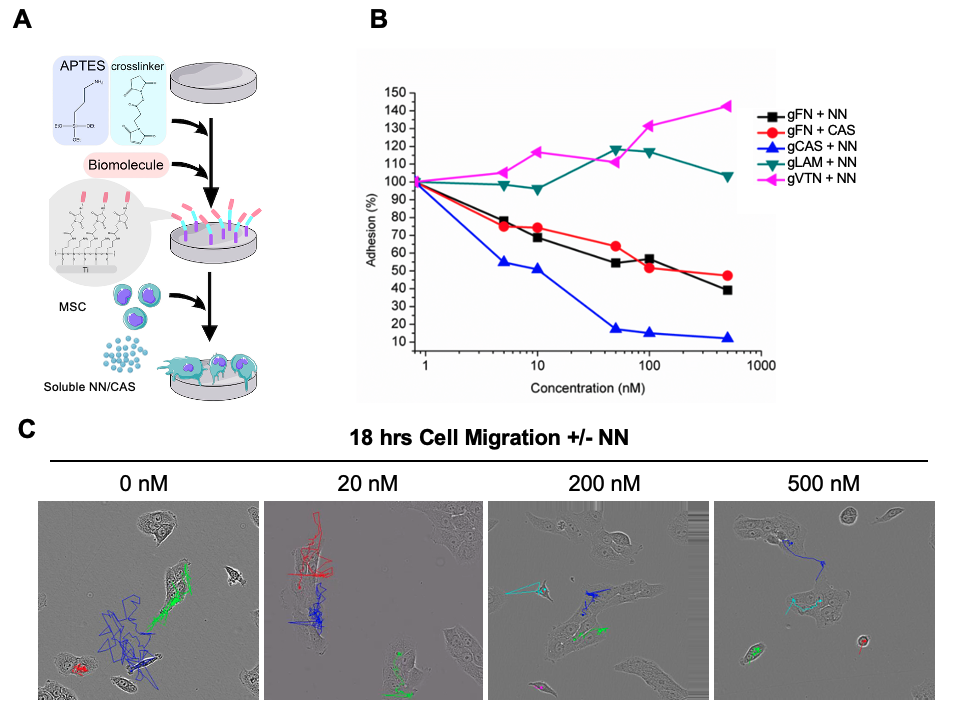
**

**Figure S7 Soluble NeoNectin inhibits α5β1-mediated cellular behaviors, related to Figure 4.**

(A) Schematic of the covalent immobilization of NeoNectin on Ti discs.

(B) Percentage of cells adhered on titanium discs Grafted with FN (gFN), the cell attachment fragment from FN (gCAS), laminin (gLAM), or vitronectin (gVTN) when co-incubated with soluble NeoNectin (NN) or soluble CAS .

(C) Trajectories of individual cells tracked over an 18 hours imaging period in presence of 0, 20, 200, and 500nM NeoNectin.

**
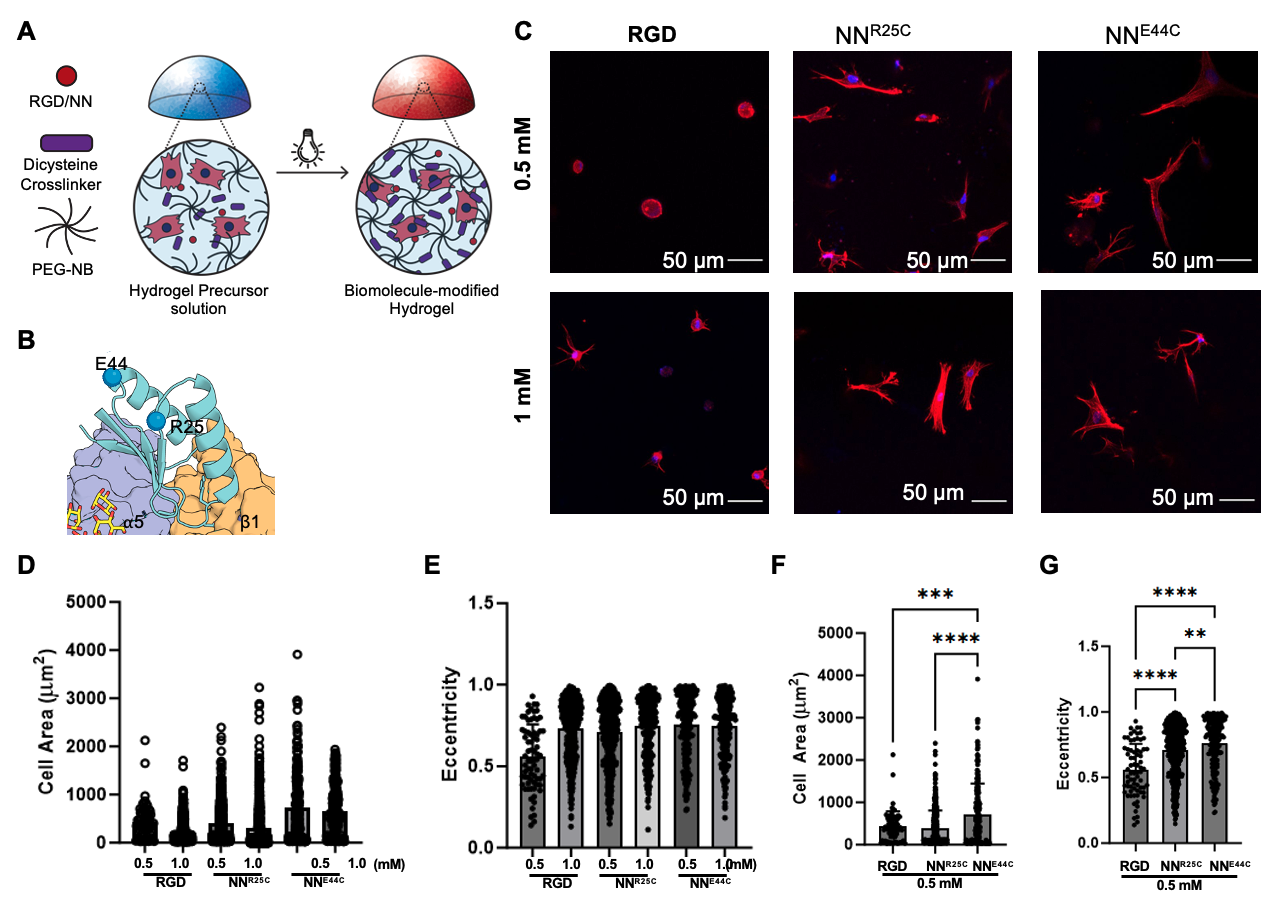
**

**Figure S8 Hydrogel modification enhances cells spreading, related to Figure 5.**

(A) Schematic representation of the radical thiol-ene-based covalent immobilization of NeoNectin in hydrogel.

(B) NeoNectin variants with cysteine mutations.

(C) Representative images of MSCs encapsulated in RGD, NN^R25C^, and NN^E44C^ modified hydrogels post 5d of culture.

(D-E) Quantification of cell spread area and eccentricity across all conditions tested.

F-G, Quantification of cell area and eccentricity in the 0.5 mM conditions, which displayed the greatest variation across all tested. Error bars = S.D. One Way ANOVA, Tukey’s Post-hoc Test. ** = p < 0.01, *** = p < 0.001, **** = p < 0.0001.

**
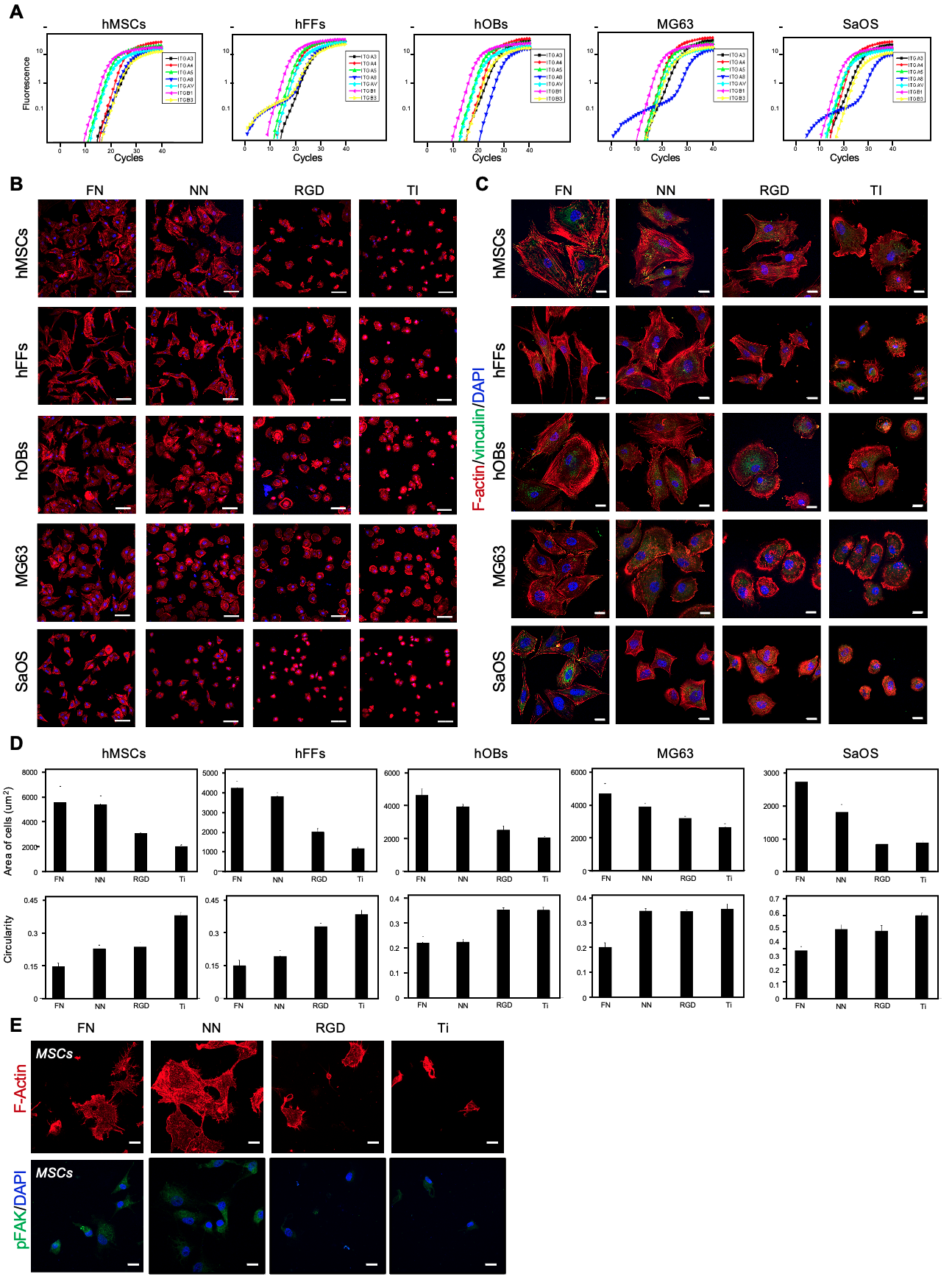
Figure S9 Modulation of cell adhesion by NeoNectin immobilized onto Ti discs, related to Figure 5.**

(A) Real-time PCR amplification plots showing the expression of different integrins in MSCs, FFs, OBs, MG63 and SaOS-2 cells.

(B) Representative immunofluorescence images of MSCs, FFs, OBs, MG63 and SaOS-2 cells after 4h of adhesion on the different functionalized Ti discs. F-actin (red), vinculin (green), and nuclei (DAPI, blue) were stained. Vinculin serves as a marker of focal adhesions. The scale bar denotes 100 µm.

(C) Representative high magnification images from B. The scale bar denotes 10 µm.

(D) Quantification of the area and circularity of cells from B.


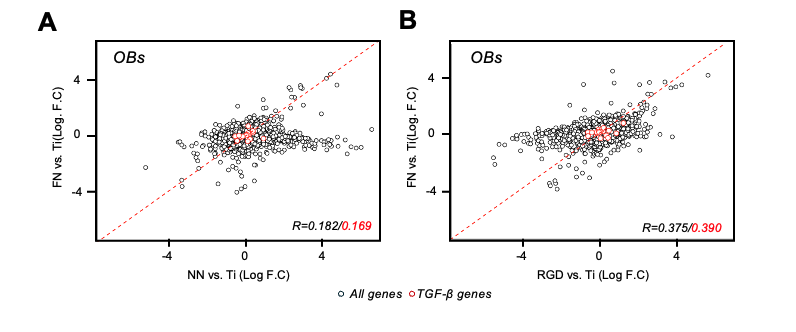


**Figure S10 Scatterplots of gene expression against bare Ti discs for FN-, NeoNectin-, and RGD-grafted Titanium discs, related to Figure 5.**

Scatterplots comparing relative gene expression (fold change of FN-,NN-, or RGD-coated over bare Ti) for OBs, related to Figure 5. FN-treated samples are on the y-axis; NN- (A) or RGD-treated (B) samples on the x-axis. Similarities of whole transcriptome (black) and TGF-β pathway genes (red) with FN-grafted samples were assessed by Pearson correlation.”


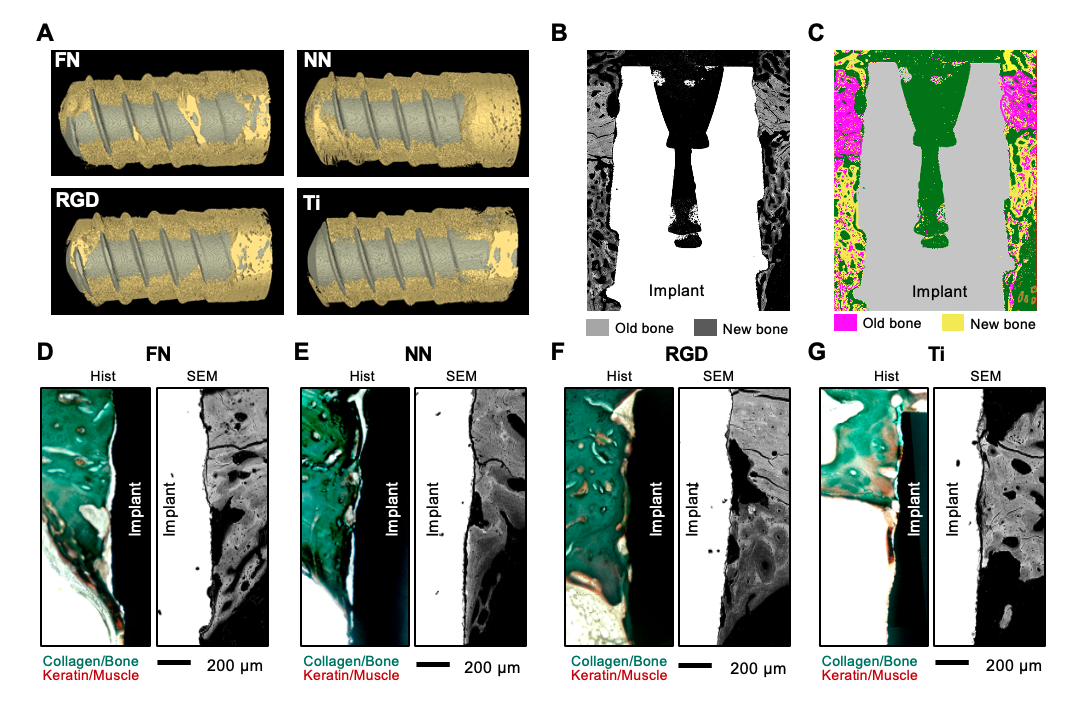
**Figure S11: NeoNectin-grafted titanium implant outperforms FN- and RGD- grafted, and bare titanium implants in stimulating implant integration and bone growth, related to Figure 6.**

(A) Representative micro-CT 3D reconstruction images showing bone (yellow) around the grafted or bare titanium implants (gray) 6 weeks post-surgery.

(B-C) Example of image processing for the calculation of new bone percentage. SEM images showing old bone in light gray and new bone in dark gray (B) were processed to show each bone type as pink and yellow, respectively.

(D-G) Representative histological staining (left) and SEM (right) images of longitudinal sections 6 weeks post-implantation showing the implants conjugated with indicated molecules inserted into the tibia of rabbits.

**Table S1. Cryo-EM Data Collection and Processing Statistics**

|  | **Extended Open**  **α5β1:NeoNectin** | **Extended Closed**  **α5β1: NN-C2** | **Extended Open**  **α5β1: NN-C2** |
| --- | --- | --- | --- |
|  |  |  |  |
|  |  |  |  |
| **EMDB** | 45655 | 46902 | 47968 |
| **PDB** | 9CKV | 9DIA | 9EF2 |
| **Magnification** | 36,000x | 36,000x | 36,000x |
| **Voltage (kV)** | 200 | 200 | 200 |
| **Electron exposure (e–/Å^2^)** | 50 | 50 | 50 |
| **Defocus range (μm)** | 1.2 - 2.0 | 1.0 - 2.0 | 1.0 - 2.0 |
| **Pixel size (Å)** | 1.122 | 1.122 | 1.122 |
| **Symmetry imposed** | C1 | C1 | C1 |
| **Initial particle images (no.)** | 728,379 | 1,150,388 | 1,150,388 |
| **Final particle images (no.)** | 72,604 | 153,759 | 69,848 |
| **Map resolution (Å)**  **Overall**  **Local Refinement** | 3.28  3.19 | 2.98  2.97 | 3.95  3.56 |
|  |  |  |  |
| **FSC threshold** | 0.143 | 0.143 | 0.143 |
| **Map resolution range (Å)**  **Overall**  **Local Refinement** | 2.92 - 17.44  2.92-9.65 | 2.58 - 39.58  2.59 - 7.86 | 3.25- 56.53  3.17- 10.21 |
|  |  |  |  |

**NN-C2: NeoNectin candidate 2*

**Table S2. Surface elemental atomic composition (%) and thickness (nm) of grafted titanium discs**

| **Groups** | **N 1s** | **Ti 2p** | **C 1s** | **O 1s** | **Si 2p** | **Thickness** |
| --- | --- | --- | --- | --- | --- | --- |
| **FN** | 8.6 ± 0.05 | 2.6 ± 0.56 | 58.5 ± 2.49 | 26.2 ± 1.41 | 4.1 ± 0.63 | 3.34 ± 0.64 |
| **NeoNectin** | 7.3 ± 0.46 | 3.3 ± 0.24 | 57.4 ± 2.50 | 27.0 ± 1.27 | 5.1 ± 0.41 | 2.68 ± 0.21 |
| **RGD** | 4.4 ± 0.25 | 3.7 ± 0.00 | 48.9 ± 0.81 | 35.2 ± 0.83 | 7.8 ± 0.32 | 2.29 ± 0.07 |
| **Ti** | 2.1 ± 0.41 | 11.6 ± 0.94 | 39.4 ± 0.10 | 45.9 ± 0.31 | 1.0 ± 0.32 | - |
